# Systematic DFT Studies on Binary Pseudo‐tetrahedral Zintl Anions: Relative Stabilities and Reactivities towards Protons, Trimethylsilyl Groups, and Iron Complex Fragments

**DOI:** 10.1002/chem.202001379

**Published:** 2020-08-17

**Authors:** Lukas Guggolz, Stefanie Dehnen

**Affiliations:** ^1^ Fachbereich Chemie and Wissenschaftliches Zentrum für, Materialwissenschaften (WZMW) Philipps-Universität Marburg Hans-Meerwein-Straße 4 35043 Marburg Germany

**Keywords:** binary Zintl anions, cluster compounds, DFT calculations, electronic structure, main group elements

## Abstract

Binary pseudo‐tetrahedral Zintl anions composed of (semi)metal atoms of the p‐block elements have proven to be excellent starting materials for the synthesis of a variety of heterometallic and intermetalloid transition metal–main group metal cluster anions. However, only ten of the theoretically possible 48 anions have been experimentally accessed to date as isolable salts. This brings up the question whether the other species are generally not achievable, or whether synthetic chemists just have not succeeded in their preparation so far. To contribute to a possible answer to this question, global minimum structures were calculated for all anions of the type (TrTt_3_)^5−^, (TrPn_3_)^2−^, and (Tt_2_Pn_2_)^2−^, comprising elements of periods 3 to 6 (Tr: triel, Al⋅⋅⋅Tl; Tt: tetrel, Si⋅⋅⋅Pb; Pn: pnictogen, P⋅⋅⋅Bi). By analyzing the computational results, a concept was developed to predict which of the yet missing anions should be synthesizable and why. Additionally, the results of an electrophilic attack by protons or trimethylsilyl groups or a nucleophilic attack by transition metal complex fragments are described. The latter yields butterfly‐like structures that can be viewed as a new form of adaptable tridentate chelating ligands.

## Graphical abstract

**More than ten**! Systematic DFT studies of all 48 binary anions of p‐block (semi)metals of periods 3–6, with 20 valence electrons and pseudo‐tetrahedral shape suggest that more than the known ten species should be generally accessible experimentally. Furthermore, the investigations illustrate the impact of the respective elemental combinations on substitution with electrophiles or nucleophiles, in comparison with isoelectronic P_4_, As_4_, or Si_4_^4−^.

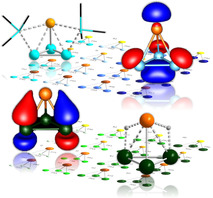

## Introduction

One century after the discovery^1^ and the first structural characterization^2^ of Zintl anions, their chemistry has become remarkably diverse.^3^ However, there are still challenges that inorganic chemists are facing while seeking for novel Zintl cluster compositions, structures, and eventually properties.

During the past two decades, it was shown that Zintl anions and their respective salts are excellent starting materials for the generation and isolation of compounds with heterometallic and intermetalloid cluster anions. While the use of homoatomic Zintl anions yields bimetallic clusters, reactions of binary Zintl anions, several of which have been known to exist with atoms of groups 13 and 14, 13 and 15, or 14 and 15, usually lead to the formation of trimetallic clusters. It was shown that the larger degree of freedom upon using binary anions as precursors is reflected in a large variety of new cluster structures and bonding modes.

In this context, binary pseudo‐tetrahedral anions that are isoelectronic with P_4_ or As_4_ are of particular interest. They show a high reactivity in cluster synthesis, but exhibit a lower overall charge than homoatomic Zintl anions Tt_4_^4−^ (Tt: Si, Ge, Sn, Pb) for elemental combinations involving groups 13 and 15 or 14 and 15 in the anions (TrPn_3_)^2−^ and (Tt_2_Pn_2_)^2−^ (Tr: triel, Tt: tetrel, Pn: pnictogen). However, not all of these elemental combinations could be experimentally accessed to date. Some of them, like the combination of Ge and Bi, yielded other anions like (Ge_4_Bi_14_)^4−^,^4^ so such combinations may systematically be unsuitable for this pseudo‐tetrahedral arrangement. To understand these findings, and to investigate the relative stabilities of these anions for predicting possible extensions of the known collection, we performed extensive and systematic computational studies on binary pseudo‐tetrahedral anions with the general formulae (TrTt_3_)^5−^, (TrPn_3_)^2−^, and (Tt_2_Pn_2_)^2−^.

Another contribution to contemporary Zintl anion chemistry is charge reduction by (element‐)organic ligand decoration, as an alternative or addition to the admixture of neutral atoms.^5^ So, in a second step, we studied the reactivity and possible functionalization of the pseudo‐tetrahedral anions with protons, trimethylsilyl groups, or organometallic substituents. We applied density functional theory (DFT) methods (*vide infra*) to simultaneously optimize geometric and electronic structures.

The results presented herein provide a deeper understanding of binary pseudo‐tetrahedral Zintl anions. Being valence isoelectronic to P_4_ in white phosphorous and As_4_ in yellow arsenic, these species can either be described by the pseudo‐element concept, also referred to as Zintl–Klemm–Busmann concept,^6^ or as a nido‐type cluster according to the Wade–Mingos rules.^7^ Since most of the anions are intrinsically disordered in the crystal structures of the according salts, this study might additionally be helpful for the interpretation of experimental data in previous and future work, and might also be helpful for the selection of potential candidates for derivatization reactions.

## Results and Discussion

### Unsubstituted binary pseudo‐tetrahedral Zintl anions

Of the herein studied pseudo‐tetrahedral Zintl anions with said elemental combinations, only examples with the general formulae (TrTt_3_)^5−^, (TrPn_3_)^2−^, and (Tt_2_Pn_2_)^2−^ have been isolated to date, which is why we focused our studies on these species. In theory, 48 element combinations should be possible. However, only ten anions have been reported, namely, (TlSn_3_)^5−^, (GaBi_3_)^2−^, (InBi_3_)^2−^, (TlBi_3_)^2−^, (Ge_2_P_2_)^2−^, (Ge_2_As_2_)^2−^, (Sn_2_Sb_2_)^2−^, (Sn_2_Bi_2_)^2−^, (Pb_2_Sb_2_)^2−^, and (Pb_2_Bi_2_)^2−^.^8^ The missing 38 species were added as calculated structures within this study.

**Figure 1:**
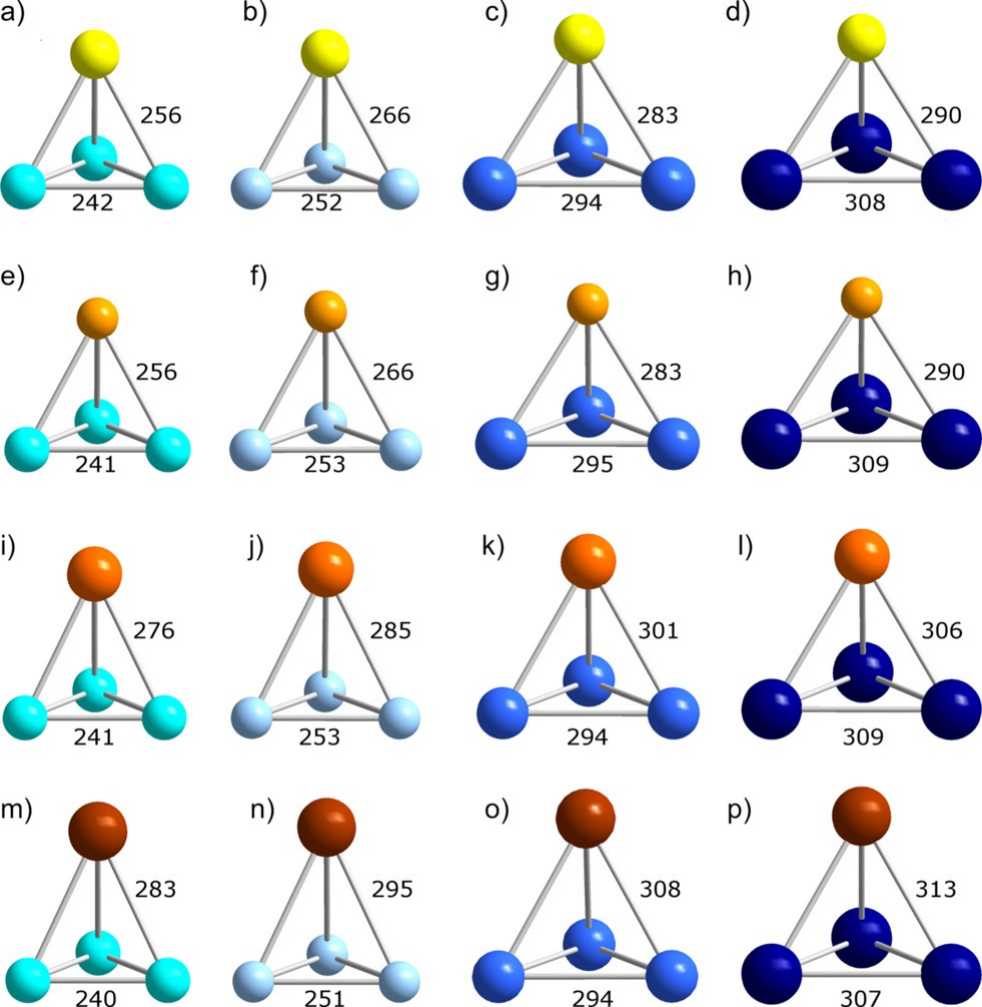
Calculated minimum structures in *C*_3v_ symmetry of all possible (TrTt_3_)^5−^ type anions: a) (AlSi_3_)^5−^, b) (AlGe_3_)^5−^, c) (AlSn_3_)^5−^, d) (AlPb_3_)^5−^, e) (GaSi_3_)^5−^, f) (GaGe_3_)^5−^, g) (GaSn_3_)^5−^, h) (GaPb_3_)^5−^, i) (InSi_3_)^5−^, j) (InGe_3_)^5−^, k) (InSn_3_)^5−^, l) (InPb_3_)^5−^, m) (TlSi_3_)^5−^, n) (TlGe_3_)^5−^, o) (TlSn_3_)^5−^, p) (TlPb_3_)^5−^ (Al: yellow, Ga: light orange, In: orange, Tl: brown, Si: turquoise, Ge: sky blue, Sn: blue, Pb: dark blue). Bond lengths are given in pm.

**Figure 2:**
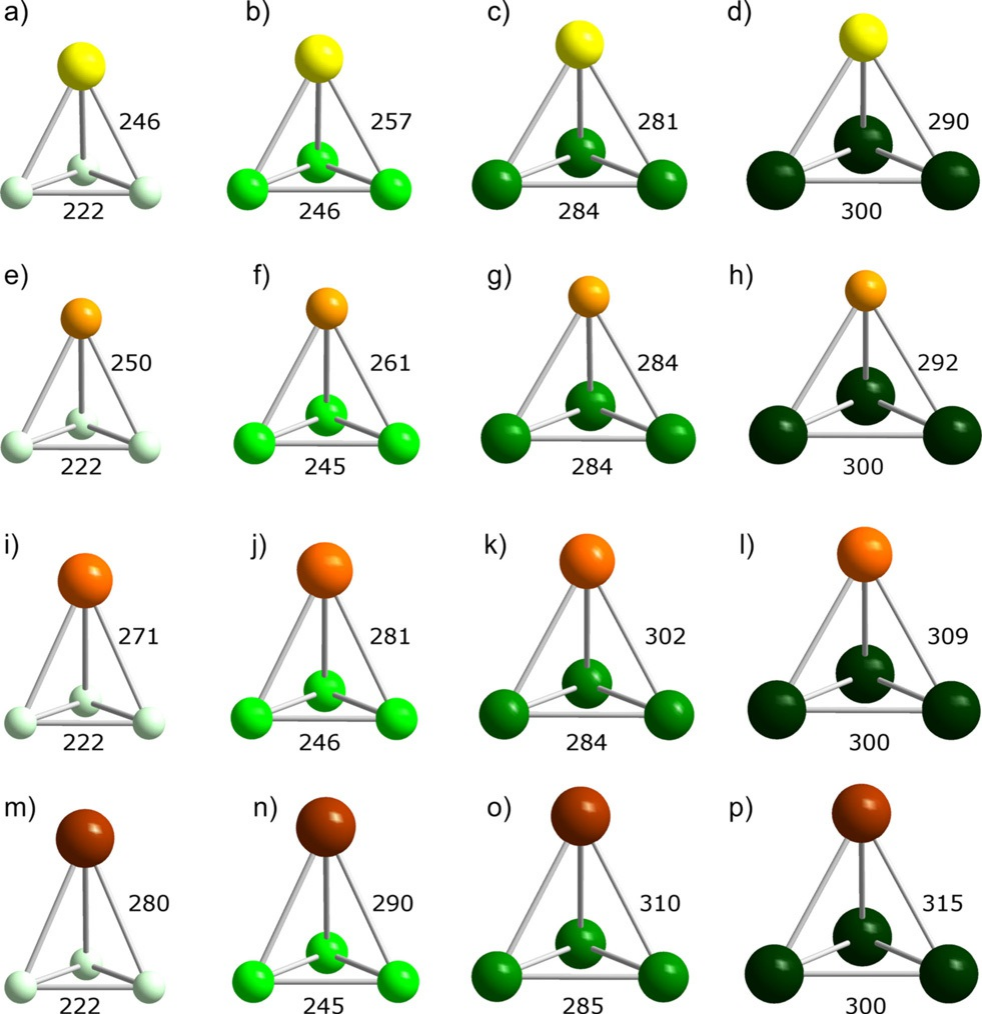
Calculated minimum structures in *C*_3v_ symmetry of all possible (TrPn_3_)^2−^ type anions: a) (AlP_3_)^2−^, b) (AlAs_3_)^2−^, c) (AlSb_3_)^2−^, d) (AlBi_3_)^2−^, e) (GaP_3_)^2−^, f) (GaAs_3_)^2−^, g) (GaSb_3_)^2−^, h) (GaBi_3_)^2−^, i) (InP_3_)^2−^, j) (InAs_3_)^2−^, k) (InSb_3_)^2−^, l) (InBi_3_)^2−^, m) (TlP_3_)^2−^, n) (TlAs_3_)^2−^, o) (TlSb_3_)^2−^, p) (TlBi_3_)^2−^ (Al: yellow, Ga: light orange, In: orange, Tl: brown, P: light green, As: bright green, Sb: green, Bi: dark green). Bond lengths are given in pm.

**Figure 3:**
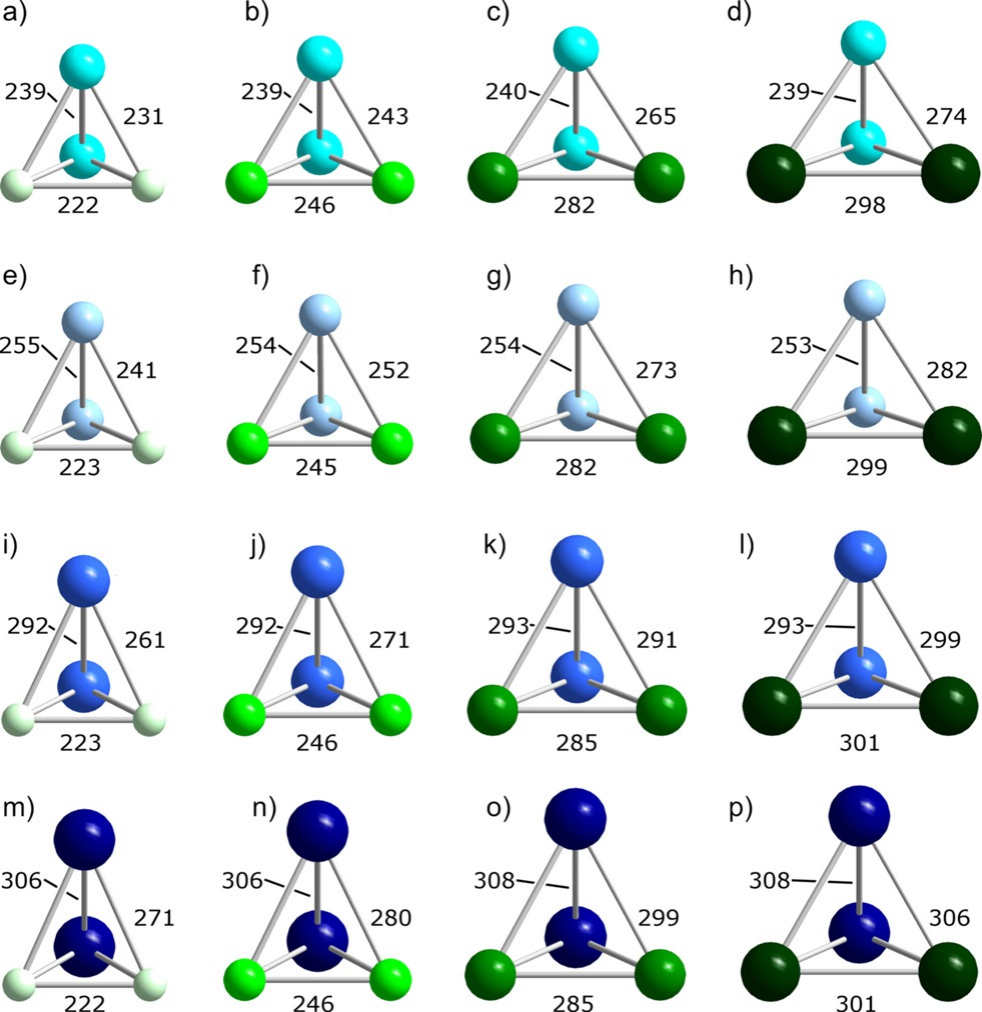
Calculated minimum structures in *C*_2v_ symmetry of all possible (Tt_2_Pn_2_)^2−^ type anions: a) (Si_2_P_2_)^2−^, b) (Si_2_As_2_)^2−^, c) (Si_2_Sb_2_)^2−^, d) (Si_2_Bi_2_)^2−^, e) (Ge_2_P_2_)^2−^, f) (Ge_2_As_2_)^2−^, g) (Ge_2_Sb_2_)^2−^, h) (Ge_2_Bi_3_)^2−^, i) (Sn_2_P_2_)^2−^, j) (Sn_2_As_2_)^2−^, k) (Sn_2_Sb_2_)^2−^, l) (Sn_2_Bi_2_)^2−^, m) (Pb_2_P_2_)^2−^, n) (Pb_2_As_2_)^2−^, o) (Pb_2_Sb_2_)^2−^, p) (Pb_2_Bi_2_)^2−^ (Si: turquoise, Ge: sky blue, Sn: blue, Pb: dark blue, P: light green, As: bright green, Sb: green, Bi: dark green). Bond lengths are given in pm.

In a first step, we performed simultaneous optimizations of the geometrical and electronic structures to find minimum structures on the potential energy surface for all known and unknown anions. It must be noted that the term “stability”, as used in the following, does not strictly refer to “thermodynamic stability” (although we find reasonable HOMO–LUMO gaps of ≥2 eV for unsubstituted pseudo‐tetrahedra, ≥1.5 eV for substituted species, and ≥1 eV for complexes; see Tables S1–S11), but to “experimental feasibility” or “experimental accessibility” throughout. We note in addition that, compared to the experimental data, most bond lengths were elongated by a few pm on average during the geometry optimization, as typical and expected for the applied methods. Nevertheless, we were able to accurately reproduce the experimental data within the error of the method (Table S12 in the Supporting Information). We therefore used the selected methods to further study all unknown binary pseudo‐tetrahedral Zintl anions. We were indeed able to find a stable minimum structure for every possible group 13/14, 13/15, and 14/15 elemental combination (see Figure 1, Figure 2, and Figure 3).

As shown in Figure 1 and Figure 2, the minimum structures of (TrTt_3_)^5−^ and (TrPn_3_)^2−^ exhibit *C*_3v_ symmetry, although originally calculated without any symmetry restrictions. These anions can hence be viewed as distorted tetrahedra with the triel atom sitting on an apex over a triangular base consisting of tetrel or pnictogen atoms, respectively. The bond lengths within the bases are in the range of bond lengths that were reported for the known homoatomic tetrahedra of (Tt_4_)^4−^ or Pn_4_, respectively.^9^ The lengths of the heteroatomic bonds vary according to the trends of the covalent radii, as expected. Figure 3 shows that the pseudo‐tetrahedral anions of the type (Tt_2_Pn_2_)^2−^ can be viewed as being composed of two homoatomic dumbbells. As expected, the deviation from an ideal tetrahedral shape is most obvious for elemental combinations with extreme differences in atomic sizes, like (Si_2_Bi_2_)^2−^ or (Pb_2_P_2_)^2−^. The heteroatomic bonds again increase as the radii of the involved atoms become larger. Tt−Tt and Pn−Pn bond lengths are in the same range as calculated for (TrTt_3_)^5−^ or (TrPn_3_)^2−^. This indicates that the ring strain in the trigonal bases does not play a significant role compared to the overall strain of the pseudo‐tetrahedral architecture.

Mulliken analyses,^10^ natural population analyses,^11^ as well as population analyses based on occupation numbers^12^ were performed to gain further insight into the electronic properties of the anions. It was shown that the negative charges are always delocalized over all four cluster atoms—in stark contrast to the formal charge assignment done by means of the pseudo‐element concept, according to which group 15 atoms are neutral, group 14 atoms are charged −1, and group 13 atoms are charged −2. This even holds for the compounds with the highest differences in electronegativity, like (InP_3_)^2−^, (TlP_3_)^2−^, or (Pb_2_P_2_)^2−^. Still, the highest partial charges, and thus the highest electron densities, are located at the more electronegative sites, indicating that the formalism of the pseudo‐element concept is oversimplifying the matter, while the trend is still correct. The results of the population analyses also showed that the distribution of the partial charges plays no significant role for the stabilities of these anions.

**Figure 4:**
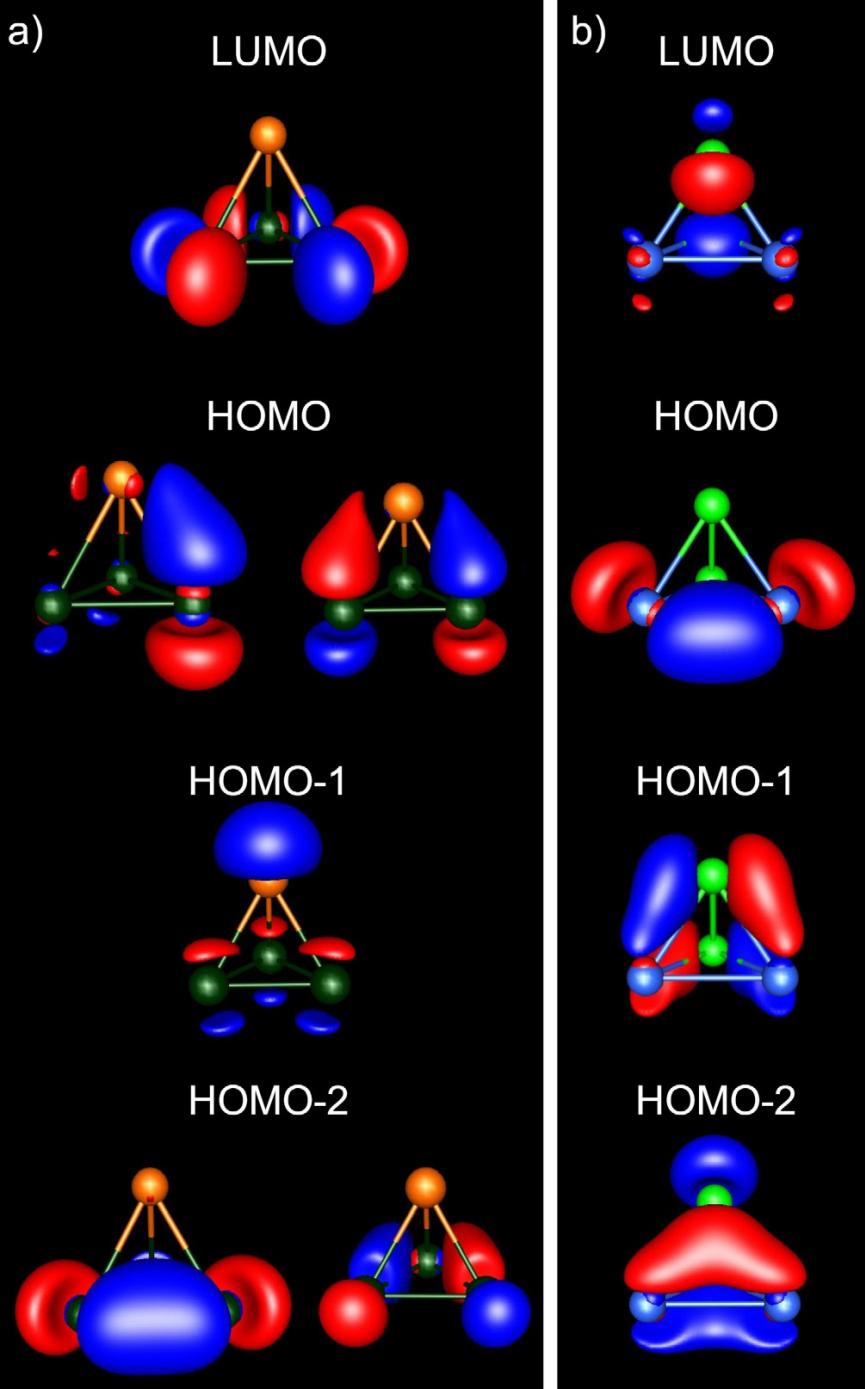
Illustration of the frontier orbitals of a) (InBi_3_)^2−^, as a representative for the anions with a 1:3 element ratio and of b) (Sn_2_As_2_)^2−^ as a representative for the anions with a 1:1 element ratio (In: orange, Bi: dark green, Sn: blue, As: bright green; contour values: ±0.05 a. u.). It must be noted that the orientation of the (Sn_2_As_2_)^2−^ anion is different from the orientation in Figure 2.

Figure 4 illustrates the frontier molecular orbitals (MOs) of the three types of anions. The MOs of (TrTt_3_)^5−^ and (TrPn_3_)^2−^ look qualitatively the same, which is due to their common 1:3 element ratio. That is except for a few group 13/14 anions, where the HOMO−1 and the HOMO−2, as well as the LUMO and the LUMO+1, are interchanged (see Figure S1 in the Supporting Information). The latter has, however, only a minor influence on the observed substitution patterns (vide infra). The lowest unoccupied molecular orbital (LUMO) of the (TrTt_3_)^5−^ type anions is always located at the triangular tetrel base. The highest occupied molecular orbital (HOMO) is doubly degenerate and extends along the heteroatomic bonds. The main contribution to HOMO−1 stems from the group 13 atom, hence essentially representing its lone pair. The doubly degenerate HOMO−2 only shows contributions of the three group 14 or group 15 atoms. As an alternative, the HOMO−2 and the LUMO may be viewed as representing the bonding (occupied) e and antibonding (unoccupied) a_1_ combination of the in‐plane tangential p‐type atomic orbitals (p‐AOs) of a hypothetical “Tt_3_^2−^” anion, hence the 3‐center‐4‐electron (3c4e) σ‐type bond of this species, which is isoelectronic with the (C_3_H_3_)^+^ cation). HOMO−1 and HOMO are based on the bonding (and occupied) a_1_ and the antibonding (and unoccupied) e representation of the orthogonal p‐AOs, hence the 2π aromatic system of the said species. The latter overlap effectively with the p‐AOs of a hypothetical “Tr^3−^” anion, which adds another 4 p‐electrons to the 10 electrons in 5 highest occupied MOs of the resulting anion. As a consequence of the combination of “Tt_3_^2−^” and “Tr^3−^”, the binary anions become electron‐precise with only minor multi‐center bonding (vide infra). For the *C*_2v_‐symmetric (Tt_2_Pn_2_)^2−^ type anions, the LUMO extends along the Pn−Pn bond, the HOMO along the Tt−Tt bond and the HOMO−1, as well as the HOMO−2, along the heteroatomic bonds.

**Figure 5:**
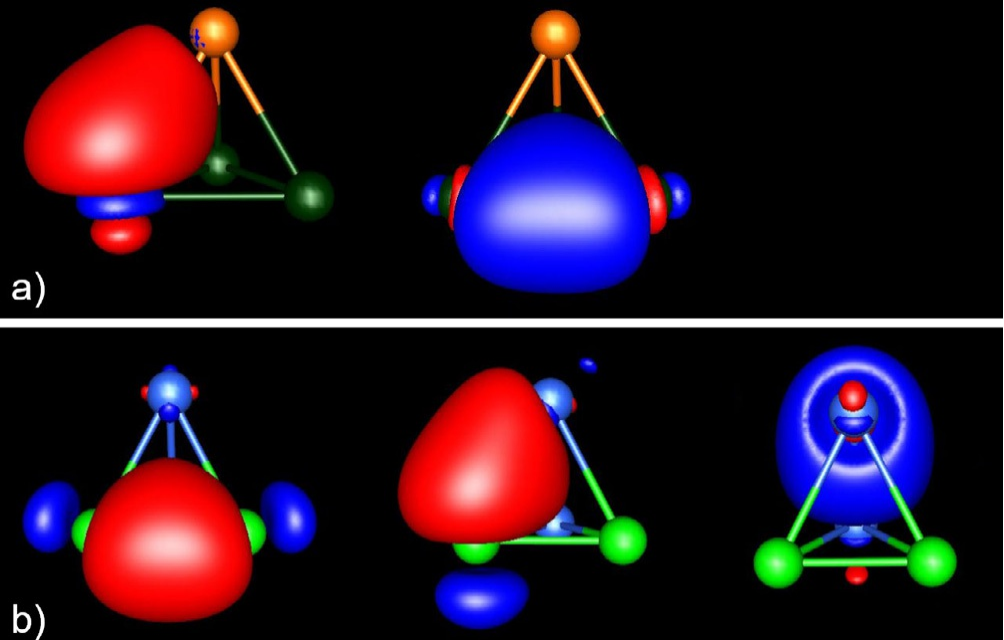
Illustration of the LMOs of the bonds of a) (InBi_3_)^2−^ and in b) (Sn_2_As_2_)^2−^ (In: orange, As: bright green, Bi: dark green, Sn: blue; contour values: ±0.05 a. u.).

To further investigate the bonding situation within these clusters, we calculated localized molecular orbitals (LMOs) according to Boys’ method.^13^ This is exemplified in Figure 5 for the bonds in (InBi_3_)^2−^ and (Sn_2_As_2_)^2−^. Since we were able to localize the MOs, the overall bonding in this polyhedra can be viewed as being based on regular 2‐center‐2‐electron (2c2e) bonds (in agreement with the pseudo‐element concept, rendering Wade–Mingos rules less appropriate), with the main contributions coming from the p‐AOs. This is in good agreement with earlier studies.^14^ For both clusters, we clearly observe a polarization of the heteroatomic bonds. Further examination via Paboon, however, also indicated additional weak multi‐center interactions, which become smaller with increasing atomic number.

We would like to note here that our findings are in agreement with the alternative way of discussing such clusters as superatoms^15^ and according to the Jellium model.^16^ As shown for the homoatomic 20 or 40 valence electron closed‐shell species [Si_4_]^4−^ or [Si_9_]^4−^ and their analogues,^17^ and also for heteroatomic superatoms like the monomeric unit of {[CuSn_5_Sb_3_]^2−^}_2_,^18^ the charge is naturally delocalized over the whole cluster in such cases, and only the total electron count matters.

From the results discussed so far, one cannot deduce why some of the binary pseudo‐tetrahedral Zintl anions could be synthesized and some could not, or why some of the anions seem to be more stable than others. While (Sn_2_Bi_2_)^2−^ has been synthesized for the first time 35 years ago and in several different salts since then,8a, 8g, 8h “(Ge_2_Bi_2_)^2−^” remains unknown to date. Another example is (GaBi_3_)^2−^, which can be synthesized,8e but readily decomposes and disproportionates into Ga^0^ and Bi_*n*_^*q*−^ polyanions.^19^ Besides that, the general claim of heteroatomic bonds to be energetically favored is challenged in some cases by their destabilization due to big differences of the covalent radii,^20^ as impressively shown on the example of the large polyanion (Ge_4_Bi_14_)^4−^, with strictly separated element types.^4^

All of the anions that have been known to date exhibit ratios of the covalent radii (Q_cr_) between 0.8 and 1.1, e.g., Q_cr_(Ga:Bi)=0.82, Q_cr_(Tl:Bi)=0.98 or Q_cr_(Ge:P)=1.12 (vide infra). Given that the different atomic sizes are critical, Q_cr_ should have an effect on the strength of the respective heteroatomic bonds within the pseudo‐tetrahedral architecture. To corroborate this, we calculated shared electron numbers (SEN) of the heteroatomic bonds, relative to the homoatomic Tt−Tt bonds in (TrTt_3_)^5−^ and relative to the Pn−Pn bonds in (TrPn_3_)^2−^ and (Tt_2_Pn_2_)^2−^, respectively, (SEN_rel_). The results that may be taken as a measure of the relative stability of the binary anions are given in Table 1.

**Table 1:** Calculated shared electron numbers (SEN)^[a]^ of the heteroatomic bonds relative to the Tt−Tt bonds (value set to 1.00) in (TrTt_3_)^5−^, and relative to the Pn−Pn bonds (value set to 1.00) in (TrPn_3_)^2−^ and (Tt_2_Pn_2_)^2−^, respectively, SEN_rel_.

(TrTt_3_)^5−^		(TrPn_3_)^2−^		(Tt_2_Pn_2_)^2−^	
	SEN_rel_		SEN_rel_		SEN_rel_
Al−Si	0.96	Al−P	0.96	Si−P	0.94
Al−Ge	0.80	Al−As	1.05	Si−As	0.89
Al−Sn	1.19	Al−Sb	1.06	Si−Sb	0.76
Al−Pb	1.24	Al−Bi	1.09	Si−Bi	0.70
Ga−Si	0.96	Ga−P	0.85	Ge−P	0.92
Ga−Ge	0.79	Ga−As	0.92	Ge−As	0.89
Ga−Sn	1.21	Ga−Sb	1.02	Ge−Sb	0.77
Ga−Pb	1.31	Ga−Bi	1.07	Ge−Bi	0.72
In−Si	0.83	In−P	0.81	Sn−P	0.97
In−Ge	0.77	In−As	0.90	Sn−As	0.98
In−Sn	0.90	In−Sb	0.79	Sn−Sb	0.92
In−Pb	0.79	In−Bi	0.81	Sn−Bi	0.88
Tl−Si	0.61	Tl−P	0.65	Pb−P	0.98
Tl−Ge	0.76	Tl−As	0.73	Pb−As	1.00
Tl−Sn	0.94	Tl−Sb	0.77	Pb−Sb	0.97
Tl−Pb	0.90	Tl−Bi	0.83	Pb−Bi	0.93

[a] It must be noted that shared electron numbers are not to be mistaken as an actual absolute measure for the bond strength; they just serve to illustrate trends.

Table 1 shows two trends for the (TrTt_3_)^5−^ and (TrPn_3_)^2−^ type anions: (a) The heteroatomic bonds become weaker for heavier triel atoms, as expected. (b) The opposite is the case as the tetrel or pnictogen atoms become heavier, that is, the Ga−Bi bond is stronger than the Tl−Bi bond, despite a seemingly unfavorable value of Q_cr_. At first glance, this seems to contradict the conclusions drawn above, according to which the difference of the covalent radii should be as small as possible. An explanation for this discrepancy can be derived from the molecular structures. Longer Tt−Tt or Pn−Pn bonds allow a closer approach of the triel atoms towards the center of the trigonal bases, similar to a close‐packed lattice: a Ga atom fits better in the space between three Bi atoms, than the Tl atom does, leading to a better overlap of the involved orbitals and to the formation of stronger bonds (see Figure 4). Anions with Q_cr_ smaller than the ideal value of 1.00 should therefore be more stable than those with Q_cr_>1.00.

This, however, does not explain why (GaBi_3_)^2−^ is actually less stable than (InBi_3_)^2−^ according to the experiments, while it should be more stable according to the data in Table 2. We obviously have two competing factors here—one being explained above. The second factor seems to be that the anion formation or its stability is generally hampered if the deviation from the ideal value of Q_cr_=1.00 becomes too large. In addition, charge distribution and the size of the anions in comparison to that of the cation of choice will definitely play a role in affecting the lattice energy and thus the formation of a respective isolable salt. As a result, anions of the type (TrTt_3_)^5−^ and (TrPn_3_)^2−^ with Q_cr_ close to 1.00 should be most stable, whereby deviations towards larger values are less tolerable than deviations towards smaller values.

**Table 2:** Ratios of covalent radii, Q_cr_, in anions (TrTt_3_)^5−^, (TrPn_3_)^2−^, and (Tt_2_Pn_2_)^2−^. Experimentally secured anions are italicized and printed in bold, dark grey letters. Anions that we predict to be synthesizable are printed in bold, black letters.

(TrTt_3_)^5−^		(TrPn_3_)^2−^		(Tt_2_Pn_2_)^2−^	
	Q_cr_		Q_cr_		Q_cr_
(AlSi_3_)^5−^	1.09	(AlP_3_)^2−^	1.13	**(Si_2_P_2_)^2−^**	**1.04**
**(AlGe_3_)^5−^**	**1.01**	**(AlAs_3_)^2−^**	**1.02**	**(Si_2_As_2_)^2−^**	**0.93**
**(AlSn_3_)^5−^**	**0.87**	**(AlSb_3_)^2−^**	**0.87**	(Si_2_Sb_2_)^2−^	0.80
**(AlPb_3_)^5−^**	**0.83**	**(AlBi_3_)^2−^**	**0.82**	(Si_2_Bi_2_)^2−^	0.75
(GaSi_3_)^5−^	1.10	(GaP_3_)^2−^	1.14	***(Ge***_***2***_***P***_***2***_***)***^***2*****−**^	***1.12***
**(GaGe_3_)^5−^**	**1.02**	**(GaAs_3_)^2−^**	**1.03**	***(Ge***_***2***_***As***_***2***_***)***^***2*****−**^	***1.01***
**(GaSn_3_)^5−^**	**0.88**	**(GaSb_3_)^2−^**	**0.88**	**(Ge_2_Sb_2_)^2−^**	**0.86**
**(GaPb_3_)^5−^**	**0.84**	***(GaBi***_***3***_***)***^**2−**^	***0.82***	(Ge_2_Bi_2_)^2−^	0.81
(InSi_3_)^5−^	1.28	(InP_3_)^2−^	1.33	(Sn_2_P_2_)^2−^	1.30
(InGe_3_)^5−^	1.18	(InAs_3_)^2−^	1.19	(Sn_2_As_2_)^2−^	1.17
**(InSn_3_)^5−^**	**1.02**	**(InSb_3_)^2−^**	**1.02**	***(Sn***_***2***_***Sb***_***2***_***)***^***2***−^	***1.00***
**(InPb_3_)^5−^**	**0.97**	***(InBi***_***3***_***)***^***2*****−**^	***0.96***	***(Sn***_***2***_***Bi***_***2***_***)***^***2*****−**^	***0.94***
(TlSi_3_)^5−^	1.31	(TlP_3_)^2−^	1.36	(Pb_2_P_2_)^2−^	1.36
(TlGe_3_)^5−^	1.21	(TlAs_3_)^2−^	1.22	(Pb_2_As_2_)^2−^	1.23
***(TlSn***_***3***_***)***^***5*****−**^	***1.04***	**(TlSb_3_)^2−^**	**1.04**	***(Pb***_***2***_***Sb***_***2***_***)***^***2*****−**^	***1.05***
**(TlPb_3_)^5−^**	**0.99**	***(TlBi***_***3***_***)***^***2*****−**^	***0.98***	***(Pb***_***2***_***Bi***_***2***_***)***^***2*****−**^	***0.99***

As mentioned above, the (Tt_2_Pn_2_)^2−^ type anions can formally be viewed as being composed of two homoatomic dumbbells. The lack of a similar stabilizing effect as for the (TrTt_3_)^5−^ and (TrPn_3_)^2−^ type anions results in these anions being much more sensitive to deviations from Q_cr_=1.00 towards smaller values. This is shown by the fact that (GaBi_3_)^2−^ with Q_cr_=0.82 was successfully synthesized, whereas (Ge_2_Bi_2_)^2−^ with Q_cr_=0.81 is unknown and seems to be systematically inaccessible. In contrast, deviations towards larger ratios (Q_cr_>1.00) seem to be less problematic, cf. the experimentally observed (Ge_2_P_2_)^2−^ anion (Q_cr_=1.12); still, the anion undergoes a re‐organization in solution to form a larger Zintl anion in the course of several days, indicating its metastability.

All of the considerations above lead us to the conclusion that a few more anions at least should be accessible. Table 2 lists all 48 anions along with their respective Q_cr_ values. Known anions are italicized and highlighted in bold and dark grey, while anions that we predict to be synthesizable are highlighted in bold.

In conclusion, the discovery or development of suitable synthetic methods seems to be critical for accessing the yet missing anions—notably, it took more than 80 years from the prediction of Bi_7_^3−^ and Bi_11_^3−^ polyanions to their isolation as salts.^19^, ^21^

### Reactivities and substitution patterns

Because of the high anionic charges of −2 and −5, respectively, salts of the pseudo‐tetrahedral binary anions are more difficult to handle in common (organic) solvents than species with lower or no charge. We therefore performed extensive studies on possible electrophilic substitution with protons and trimethylsilyl (TMS) groups, and on possible nucleophilic substitutions with organometallic substituents, in order to reduce the cluster charge, and thereby modify properties like solubility and reactivity. As none of these attempts proved possible for the herein discussed binary anions in experimental work so far, we aimed at examining the effects of substituents on the molecular structures by theoretical work. We limited our efforts to structures with two substituents, neutral (TrPn_3_R_2_), (Tt_2_Pn_2_R_2_), as well as anionic (TrTt_3_R_2_)^3−^ and (Tt_2_Pn_2_R_2_)^2−^ (in case of organometallic substituents).

While first theoretical studies of a singly protonated P_4_ tetrahedron suggested the proton to be located at an apex of the molecule,^22^ protonation of the tetrahedral edges was predicted to be energetically favored later on.^23^ This substitution pattern was recently verified experimentally, and by means of new DFT and coupled‐cluster calculations, for the first known protonated variants of tetrahedral 20 valence electron species, [P_4_(μ‐H)]^+[24a]^ and [Si_4_(μ‐H)]^3−^,24b as well as a protonated unit used as ligand to ZnPh_2_ in [(μ‐H)(η^2^‐Ge_4_)ZnPh_2_]^3−^.24c Additionally, Scheschkewitz and co‐workers recently reported (Si_5_R_4_)^2−^. This anion can be interpreted as an Si_4_^4−^ tetrahedron with two substituents (thus reducing the overall charge) and an additional SiR_2_ moiety acting as an electrophile and bridging one of the Si−Si bonds of the underlying tetrahedral structure motif.24d In our theoretical study, we added two protons to the anions (in order to compensate for all charges of the (TrPn_3_)^2−^ and (Tt_2_Pn_2_)^2−^ species), and explored all possible protonation sites of the resulting species.

Anions of the type (TrTt_3_)^5−^ and (TrPn_3_)^2−^ show the same preferred protonation pattern. As expected, the hydrogen atoms bridge two heteroatomic bonds by involving the doubly degenerate HOMO (see Figure 4) in the energetically favored isomers, resulting in *C*_s_‐symmetric molecules. The bridge is most asymmetric for the two lightest homologues, (AlSi_3_H_2_)^3−^ and (AlP_3_H_2_), where the H atoms are much closer to the Al atoms.

**Figure 6:**
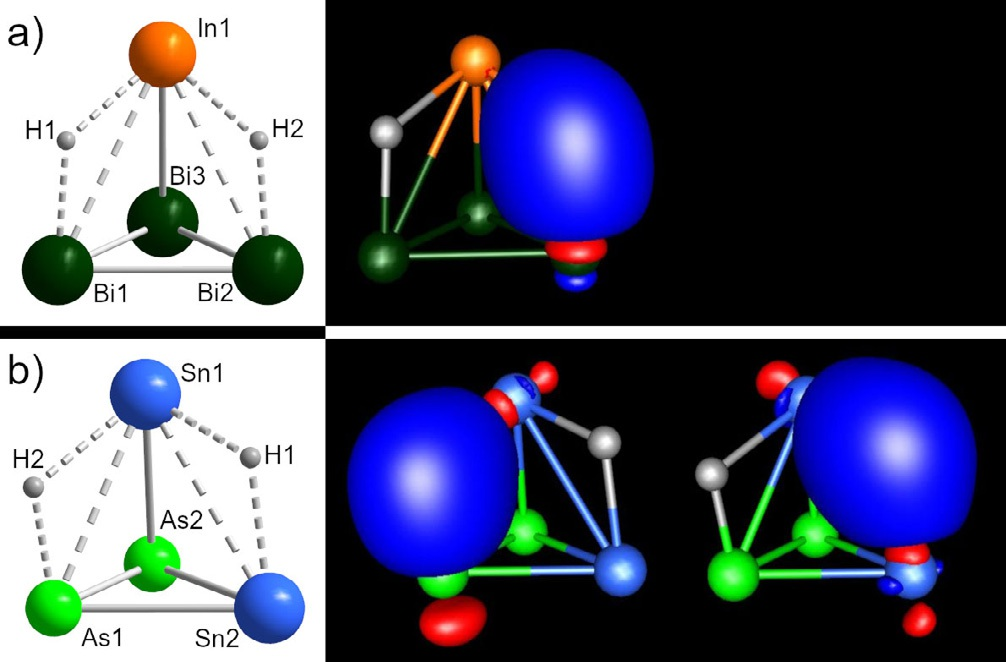
a) Calculated minimum structure of (InBi_3_H_2_) and illustration of one of the two LMOs representing the two 3c2e bonds; b) Calculated minimum structure of (Sn_2_As_2_H_2_) and LMOs representing the two 3c2e bonds (In: orange, As: bright green, Bi: dark green, Sn: blue, H: grey; contour values: ±0.05 a. u.). It must be noted that the orientation of the (Sn_2_As_2_H_2_) molecule is different from the orientation of the parent (Sn_2_As_2_)^2−^ anion in Figure 2.

For the (Tt_2_Pn_2_)^2−^ type anions, bridging of the Tt−Tt bond and one of the heteroatomic bonds is most favorable, which is realized by involvement of HOMO and HOMO−1. In all cases, the (μ‐H)‐bridged bonds are significantly elongated, by 8–10 % for all three cluster types (see Figure 6 for (InBi_3_H_2_) and (Sn_2_As_2_H_2_) as examples). This is in perfect agreement with the recent experimental findings for the isoelectronic species mentioned above.

The presence of 3‐center‐2‐electron (3c2e) bonds upon μ‐H‐bridging is supported by corresponding SEN values, and the 3c2e bonds become stronger for values of Q_cr_ close to 1.00. They are hence the weakest (on average) for clusters (TrPn_3_H_2_). Table 3 lists corresponding data for (InBi_3_H_2_) and (Sn_2_As_2_H_2_) as examples.

**Table 3:** Calculated bond lengths and SEN as well as 3‐center‐SEN (3cSEN) values for (InBi_3_H_2_) and (Sn_2_As_2_H_2_); for the *C*_s_‐symmetric anion (InBi_3_H_2_), only half of the otherwise equivalent bonds are listed.

	calcd/pm	SEN	3cSEN
(InBi_3_H_2_)			
In1−Bi1	343	0.19	–
In1−H1	216	0.41	–
Bi3−H1	193	0.87	–
In1−H1−Bi3	–	–	0.11
In1−Bi3	307	0.78	–
Bi1−Bi2	297	0.96	–
Bi1−Bi3	296	1.01	–

(Sn_2_As_2_H_2_)			
Sn1−Sn2	318	0.74	–
Sn1−H1	195	0.85	–
Sn2−H1	198	0.80	–
Sn1−H1−Sn2	–	–	0.40
Sn1−As1	297	0.70	–
Sn1−H2	211	0.56	–
As1−H2	163	0.98	–
Sn1−H2−As1	–	–	0.33
Sn1−As2	263	1.23	–
Sn2−As1	272	1.03	–
Sn2−As2	271	1.08	–
As1−As2	246	1.14	–

NPA and Mulliken analyses illustrate the impact of the protonation on the electronic structures. While the negative charge was relatively evenly distributed over all four atoms in the naked anions, the three atoms that are involved in the bonds to the two hydrogen atoms (e.g., In1, Bi1, and Bi2 in Figure 6 a) have positive partial charges now. In turn, the unsubstituted (semi)metal atom (e.g., Bi3 in Figure 6 a) and both hydrogen atoms are partially negatively charged in the overall neutral cluster. In accordance with their larger electronegativity as compared to any of the p‐block (semi)metals, the hydrogen atoms thus undergo an umpolung towards a hydridic character. As illustrated in Figure 6, 3c2e bonds involving heteroatomic tetrahedral edges are slightly polarized towards the more electronegative (semi)metal atom. For the series of anions (TrTt_3_H_2_)^3−^, the negative charge is delocalized over all atoms. Yet, the largest electron density is also localized at the H atoms and the (unsubstituted) tetrel atom.

**Figure 7:**
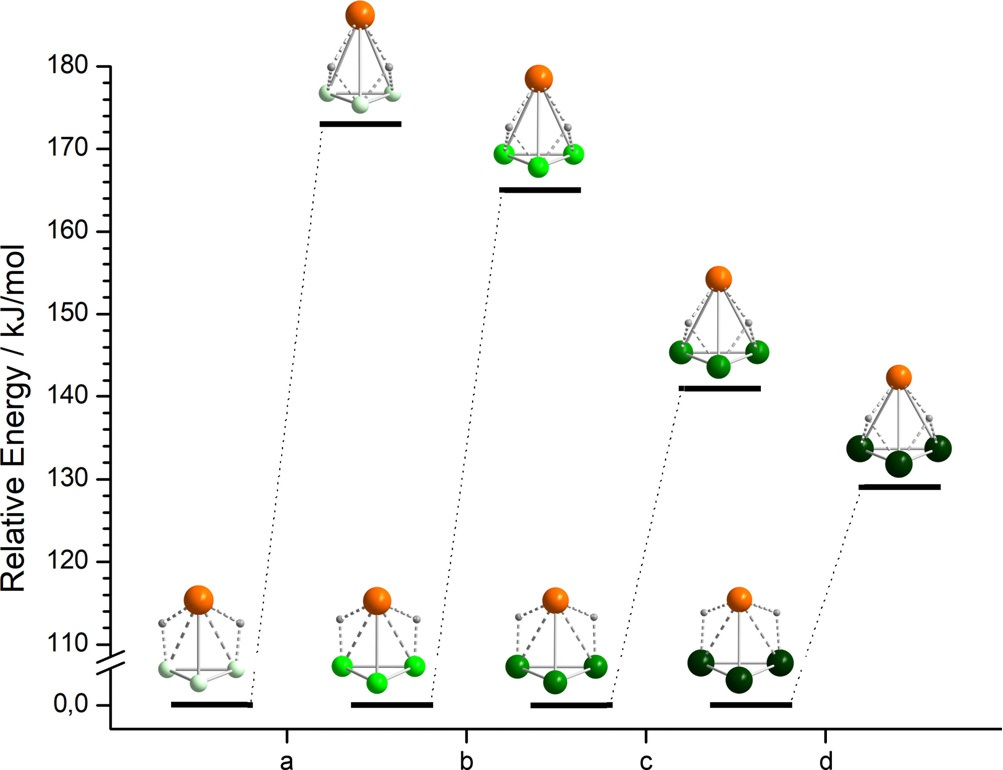
Energies of clusters with the H atoms bridging tetrahedral faces, relative to their global minimum structures with the H atoms bridging tetrahedral edges. a) {InP_3_(μ_3_‐H)_2_} vs. {InP_3_(μ‐H)_2_}, b) {InAs_3_(μ_3_‐H)_2_} vs. {InAs_3_(μ‐H)_2_}, c) {InSb_3_(μ_3_‐H)_2_} vs. {InSb_3_(μ‐H)_2_}, d) {InBi_3_(μ_3_‐H)_2_} vs. {InBi_3_(μ‐H)_2_} (In: orange, P: light green, As: bright green, Sb: green, Bi: dark green, H: grey).

Geometry optimizations with the H atoms being forced into a position over the trigonal faces were done to study stability trends. The molecular structures relaxed into local minima that were significantly higher in energy than the structures exhibiting edge‐bridging, as illustrated in Figure 7 for the series (InPn_3_H_2_) with Pn: P, As, Sb, Bi. The energy differences with respect to the global minimum decreases in the order Pn: P>As>Sb>Bi. Hence, the stabilization of the edge‐bridged isomer is more significant for underlying pseudo‐tetrahedra with larger size differences of the (semi)metal atoms, thus larger Q_cr_ in the given series.

In case of the clusters (Tt_2_Pn_2_H_2_), HOMO−2 contributes to the bond, which has a stabilizing effect. Nevertheless, the difference between the global and the local minimum is still large, e.g., 97 kJ mol^−1^ for (Sn_2_As_2_H_2_).

While protonating the binary pseudo‐tetrahedral anions always yields clusters with bridged edges, the picture becomes more complex for the (hypothetical) addition of trimethylsilyl (TMS) groups. After the geometry optimizations, we find three different (dominant) substitution patterns for clusters {TrTt_3_(SiMe_3_)_2_}^3−^, {TrPn_3_(SiMe_3_)_2_} and {Tt_2_Pn_2_(SiMe_3_)_2_}, respectively. These follow a continuous trend from a preference of edge‐bridging to a preference of terminal bonding, which can be put down to the anions’ different tendency to form efficient 3c2e bonds.

The derivatization of homoatomic, tetrahedral main group compounds with four alkylsilyl groups yielding tetrahedrane‐like structures was previously reported.^25^ The steric demand of four alkylsilyl groups results in the preference of terminal bonding over edge‐bridging. In {Tl_4_(C{SiMe_3_})_4_}, for instance, the alkylsilyl groups are tilted sideways.25d This, however, is more likely due to the steric demand of the substituents, than being an indication for a tendency to forming 3c2e bonds, as we see them in the clusters studied herein (vide infra).

**Figure 8:**
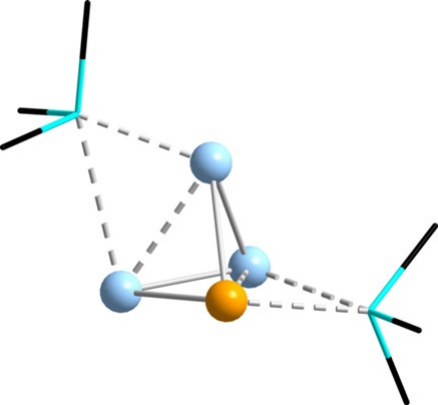
Calculated minimum structure of {GaGe_3_(SiMe_3_)_2_}^3−^ as a typical example for anions of the type {TrTt_3_(SiMe_3_)_2_}^3−^ (Ga: light orange, Ge: sky blue, Si: turquoise, C: black, H atoms are omitted for clarity).

Anions of the type {TrTt_3_(SiMe_3_)_2_}^3−^ prefer edge‐bridging under formation of two 3c2e bonds. Notably, only here, the above mentioned interchanging of HOMO−1 and HOMO−2 in anions of the type (TrTt_3_)^5−^ plays a role: in cases, in which these MOs are interchanged, the bridging involves one heteroatomic bond and one of the opposing homoatomic bonds (see Figure 8 for {GaGe_3_(SiMe_3_)_2_}^3−^ as an example), with the exception of {InGe_3_(SiMe_3_)_2_}^3−^, in which two homoatomic bonds are bridged. The other order of MOs, however, leads to bridging of two heteroatomic bonds instead (see Figure S2). The relative energy difference between both isomer types is smallest for anions comprising heaviest atoms, such as {TlPb_3_(SiMe_3_)_2_}^3−^. In all cases, addition of TMS groups causes elongation of the involved bonds, by 4–10 %. This elongation becomes less prominent for values of Q_cr_≈1.00, irrespective of the observed substitution pattern.

**Figure 9:**
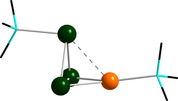
Calculated minimum structure of {InBi_3_(SiMe_3_)_2_} as a typical example for molecules of the type {TrPn_3_(SiMe_3_)_2_} (In: orange, Bi: dark green, Si: turquoise, C: black, H atoms are omitted for clarity).

Due to the larger differences of the electronegativity of the involved atoms, clusters of the type {TrPn_3_(SiMe_3_)_2_} tend the least to forming 3c2e bonds, like discussed for the protonated species. Here, the preferred substitution pattern includes two terminal bonds, with one TMS group bonded to the triel atom, and the other one bonded to one of the pnictogen atoms (see Figure 9 for {InBi_3_(SiMe_3_)_2_} as an example). The heteroatomic bonds in the 4‐vertex units are elongated by up to 10 % (most distinctly for heaviest atoms, while lighter homologues show no or only slight elongations). In this conformation, the two TMS groups are the furthest apart of all examples discussed herein. We recognize an exception from this pattern only for {InP_3_(SiMe_3_)_2_} and for the sub‐series including the heaviest triel thallium, {TlPn_3_(SiMe_3_)_2_} (with Pn: P, As, Sb; see Figure S3), where the TMS groups are bonded to two pnictogen atoms. We ascribe this to the fact that both In−Si and Tl−Si bonds are significantly disfavored in comparison with Pn−Si bonds, owing to the larger differences in covalent radii.

**Figure 10:**
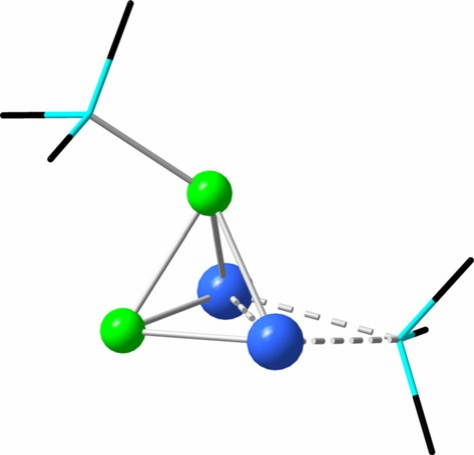
Calculated minimum structure of {Sn_2_As_2_(SiMe_3_)_2_} as a typical example for molecules of the type {Tt_2_Pn_2_(SiMe_3_)_2_} (Sn: blue, As: bright green, Si: turquoise, C: black, H atoms are omitted for clarity).

The silylation pattern for clusters of the type {Tt_2_Pn_2_(SiMe_3_)_2_} finally represents a combination of the aforementioned cases: Here, we find the Tt−Tt bond to be bridged, thereby involving the HOMO of the naked anion under formation of a 3c2e bond. The Tt−Tt bond lengths are again elongated by up to 10 %, depending on the respective Pn atoms, with the largest effect observed for the heaviest Pn atoms, again. The second TMS group is bonded as a terminal substituent to one of the Tt atoms, or to one of the Pn atoms, depending on the similarity of the covalent radii of the respective Tt or Pn atoms and the Si atoms of the terminal TMS group (Si_TMS_; see Figure 10 for {Sn_2_As_2_(SiMe_3_)_2_} as an example). In the two heaviest homologues, {Pb_2_Sb_2_(SiMe_3_)_2_} and {Pb_2_Bi_2_(SiMe_3_)_2_}, an additional bond is formed between one of the Pb atoms and the Si_TMS_ atom (see Figure S4). For this class of clusters, silicon atoms cause differences again: all species of the sub‐series {Si_2_Pn_2_(SiMe_3_)_2_} (with Pn: P, As, Sb, Bi) prefer another substitution pattern. Here, both TMS groups are bonded to the two Si atoms of the former pseudo‐tetrahedron, thereby forming a chain‐like Si_4_ moiety (Si−Si: 222–236 pm, see Figure S5).

In summary, the attachment of two protons to pseudo‐tetrahedral, binary anions of p‐block (semi)metals leads to mostly hydridic substituents. The substitution patterns for substitutions with TMS groups are different but at the same time characteristic for the chosen combination of main group elements, with some exceptions for subseries involving the lightest (Si) or heaviest (Tl) congener(s) of the involved group(s).

The calculations discussed so far addressed the electrophilic attack of binary anions. While the mono‐protonation of Si_4_^4−^ to form [Si_4_(μ_2_‐H)]^3−^ was reported,24c this has been the only example to date involving H^+^ as an electrophile, and a corresponding result remains elusive for any other tetrahedral Zintl anions. The attachment of electrophilic transition metal complex fragments like (MesCu)^+^, (ZnPh)^+^, or Zn^2+^ to a 4‐vertex anion has also been unknown for binary anions involving atoms from different main groups, but it was reported for tetrahedral anions of the type Tt_4_^4−^ (including Si/Ge mixtures).24c, 26, 27 Binary anions have so far shown to readily undergo cluster fragmentation and re‐arrangement instead, which seems to be induced or catalyzed by the transition metal atoms, thereby yielding other beautiful heterometallic and intermetalloid cluster structures.^27^

To the best of our knowledge, a nucleophilic attack towards any Zintl anion has not yet been reported until today,^27^ which sounds reasonable at first glance, owing to the presence of negative charges on the surface of the anionic molecules. However, it was previously shown that P_4_ or As_4_ can be activated via nucleophilic attack by transition metal complexes to form butterfly‐like moieties. The first compound reported to emerge from such reactions was [{Cp’’Fe(CO)_2_}_2_(μ:η^2:2^‐P_4_)] (Cp’’: η^5^‐C_5_H_3_ *t*Bu_2_).^28^ More recently, Scheer and co‐workers used [{Cp’’’Fe(CO)_2_}_2_(μ:η^2:2^‐Pn_4_)] (Pn: P, As; Cp’’’: η^5^‐C_5_H_2_ *t*Bu_3_) to show that such moieties can subsequently act as chelating ligands for Lewis acidic species, like the cationic complex fragment [Cu(NCMe)]^+^.^29^

Inspired by this work, and beyond the background that the binary Zintl anions possess the same electron count and very similar frontier orbitals, we tried to expand this concept to (hypothetic) species with binary cluster cores, [{CpFe(CO)_2_}_2_(μ:η^2:2^‐Tt_2_Pn_2_)]^2−^ (Tt: Si, Ge, Sn, Pb; Pn: P, As), hence based on pseudo‐tetrahedral species, in which two of the P or As atoms were replaced with tetrel atoms. To reduce the computational effort, we used the smaller Fp substituent (Fp: CpFe(CO)_2_^⋅^; Cp: η^5^‐C_5_H_5_) and performed geometry optimizations for the resulting molecules.

Since the LUMO of the naked anions expands along the respective Pn–Pn edge, a nucleophilic attack addresses the Pn atoms under cleavage of said edge. The Fp substituents then form terminal Fe−Pn bonds, resulting in the desired butterfly‐like structures. Our hypothesis was that the formal replacement of two of the pnictogen atoms by tetrel atoms would notably influence the electronic situation at the pnictogen atoms in the bridgehead positions. We expected to observe heterometallic chelating ligands with tunable properties at the pnictogen atoms, generally suitable for the tailored coordination of various Lewis acids.

**Figure 11:**
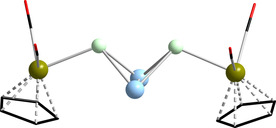
Calculated minimum structure of [{CpFe(CO)_2_}_2_(μ:η^2:2^‐Ge_2_P_2_)]^2−^ as a typical example for molecules of the type [{CpFe(CO)_2_}_2_(μ:η^2:2^‐Tt_2_Pn_2_)]^2−^ (Ge: sky blue, P: light green, Fe: dark yellow, O: red, C: black, H atoms are omitted for clarity).

The optimized structure of [{CpFe(CO)_2_}_2_(Ge_2_P_2_)]^2−^, as an example of the resulting type of anionic molecules, is shown in Figure 11. In contrast to the results obtained by the Scheer group, where the Cp’’’ groups are turned sideways and in opposite directions with respect to the “butterfly” orientation, both of the Cp ligands are orientated in the same way, yet away from the open “butterfly” edge. This difference is most likely due to the smaller steric demand of the Fp moieties in the calculated species. Table 4 summarizes relevant structural and electronic data.

**Table 4:** Pn⋅⋅⋅Pn distances, corresponding SEN values, and partial charges at the Pn atoms, as well as dihedral angles within the anions [{CpFe(CO)_2_}_2_(μ:η^2:2^‐Tt2Pn2)]^2−^ (Tt: Si, Ge, Sn, Pb; Pn: P, As).

	Si	Ge	Sn	Pb
P⋅⋅⋅P/pm	289	293	307	314
SEN (P⋅⋅⋅P)	0.19	0.18	0.15	0.14
NPA (P)	−0.56	−0.60	−0.73	−0.77
Mulliken (P)	−0.41	−0.59	−0.68	−0.72
P−Tt−Tt−P/°	91.1	90.2	88.0	87.2
As⋅⋅⋅As/pm	304	307	319	324
SEN (As⋅⋅⋅As)	0.17	0.18	0.17	0.15
NPA (As)	−0.45	−0.48	−0.61	−0.64
Mulliken (As)	−0.18	−0.34	−0.43	−0.48
As−Tt−Tt−As/°	90.4	89.4	87.0	86.1

The Pn⋅⋅⋅Pn distances become larger with increasing atomic number of the tetrel atoms. This is accompanied by smaller SEN values, which can be viewed as a very rough approximation of the trend of the remaining electron density between the two P or As atoms. The dihedral angles stay relatively constant, between 88° and 90° on average. NPA and Mulliken analyses further showed that the pnictogen atoms are partially negatively charged, while the tetrel atoms exhibit a positive partial charge. In the homoatomic analogues, [{CpFe(CO)_2_}_2_(P_4_)] and [{CpFe(CO)_2_}_2_(As_4_)], the partial charge distribution is exactly the opposite (hence questioning the equivalence of the “nucleophilic attack” in these two cases). The negative partial charge at the bridgehead sites increases as the difference in electronegativity between the two main group elements gets larger. Hence, also the softness of the respective pnictogen (donor) atom, according to the Pearson concept, increases. Therefore, these anions should be less suitable as ligands for electrophiles than the homoatomic reference clusters, which may explain the lack of experimental evidence so far.

**Figure 12:**
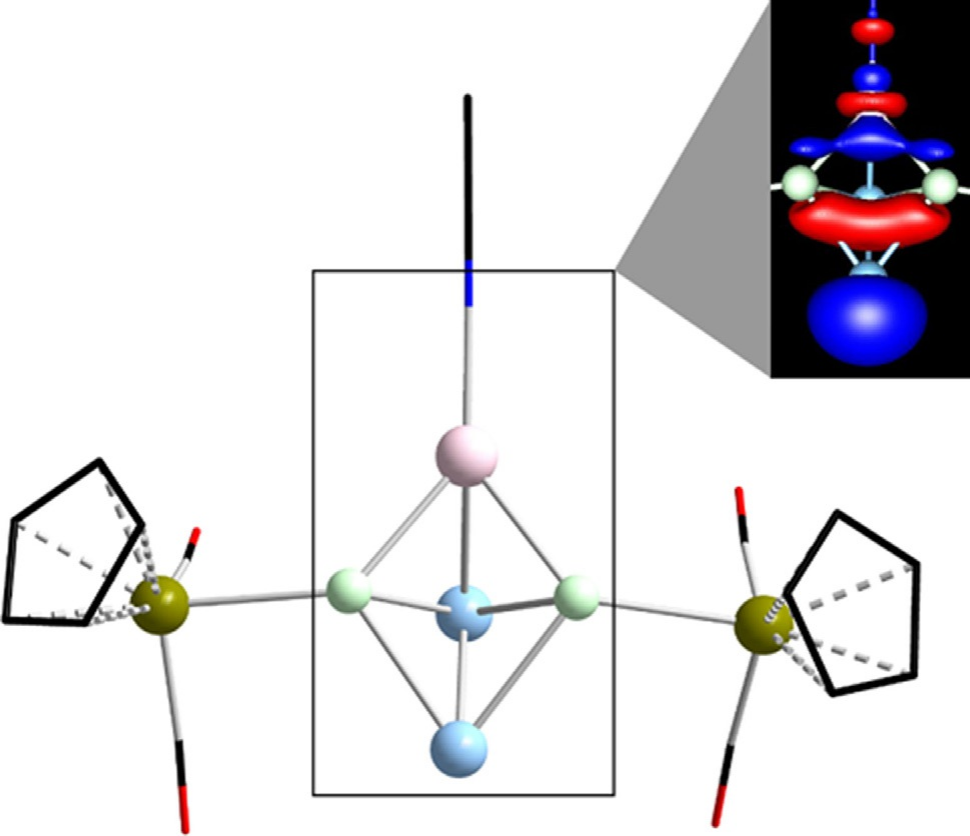
Calculated minimum structure of [Cu(NCMe)(Ge_2_P_2_{CpFe(CO)_2_}_2_)]^−^, and a cutout of the contour plot of the HOMO as the relevant bonding orbital (Ge: sky blue, P: light green, Cu: pink, Fe: dark yellow, O: red, N: blue, C: black, H atoms are omitted for clarity; contour values: ±0.05 a. u.).

To check this hypothesis, we added a [Cu(NCMe)]^+^ fragment to the butterfly‐shaped moieties in silico, and performed geometry optimizations for the anions [Cu(NCMe)(Tt_2_Pn_2_{CpFe(CO)_2_}_2_)]^−^ (Tt: Si, Ge, Sn, Pb; Pn: P, As). Indeed, the results are slightly different from the experimental findings for the homoatomic phosphorous or arsenic analogues, where the [Cu(NCMe)]^+^ fragment is pointing away from the P_4_ or As_4_ unit in an orientation perpendicular to two of the Pn–Pn edges. Here, we always find the [Cu(NCMe)]^+^ fragment to be tilted sideways, thus forming an additional Tt−Cu bond. Population analyses showed the strength of these bonds to be roughly of the same order of magnitude as for the other Pn−Fe and the Pn−Cu bonds, thus corroborating the Lewis‐basic character of the respective chelating ligand. Further back‐donation from the transition metal complex fragment into the LUMO of the ligand was not observed. The calculated minimum structure of [Cu(NCMe)(Ge_2_P_2_{CpFe(CO)_2_}_2_)]^−^, as an example of the whole series, is displayed in Figure 12, along with a cutout of the illustration of its HOMO. Figure 12 also shows that the newly formed Tt−Cu bond results from an interaction of the Cu atom's d${}_{z{}^{2}}$ atomic orbital with HOMO−4 of the chelating [{CpFe(CO)_2_}_2_(Ge_2_P_2_)]^2−^ moiety. This HOMO−4 is mainly located at the tetrel atoms and between them (see also Figure S6), thus rendering the formation of said Tt−Cu bond energetically favorable.

A comparison of the absolute energies shows that the hypothetical reactions of the respective butterfly‐like anions with a [Cu(NCMe)]^+^ fragment are highly exoenergetic (see Table S13). Furthermore, we found the P‐containing species slightly favored compared to the As‐containing analogues. This can again be explained by the Pearson concept and is in agreement with our previous results.

To verify the observed conformation to be preferred, we forced the [Cu(NCMe)]^+^ fragment into a perpendicular position by symmetry (*C*_2v_), and calculated the absolute energies of the corresponding isomers for all elemental combinations studied in this section. We found these structures to be local minima on the potential hypersurface within the given symmetry restrictions. Their total energies are between 22 kJ mol^−1^ and 82 kJ mol^−1^ above the total energies of the isomers with tilted [Cu(NCMe)]^+^ moieties, depending on the elemental combination of the underlying binary “butterfly” core. In the global minimum structures, the coordination sphere around the Cu atom is thus not trigonal planar as in the P_4_‐based and As_4_‐based structures, but strongly distorted “tetrahedral”. Hence, the [{CpFe(CO)_2_}_2_(μ:η^2:2^‐Tt_2_Pn_2_)]^2−^ type anions do not only act as bidentate, but as tridentate chelating ligands. This and the increased softness of the respective pnictogen atoms, due to their higher negative partial charge, suggest that these anions are more suitable as ligands for softer Lewis acids that tend to tetra‐coordination, such as Hg^2+^, Pt^2+^ or Ag^+^, which will be studied in future work.

## Conclusions

In summary, we presented calculated global minimum structures for all binary pseudo‐tetrahedral Zintl anions of the type (TrTt_3_)^5−^, (TrPn_3_)^2−^, and (Tt_2_Pn_2_)^2−^, composed of p‐block (semi)metals. We described structural trends, and found possible answers to the question, why some of these cluster anions seem to be systematically elusive in experimental work. At the same time, our findings allow to predict that some of the yet not isolated species should be generally accessible.

Furthermore, we studied the effect of substitution with protons or trimethylsilyl groups, and we discussed the behavior of these anions upon substitution with nucleophiles and their possible applicability as (tridentate) chelating ligands for Lewis‐acidic transition metal cations.

The findings presented herein might be of help for synthetic chemists (including ourselves) and their approaches towards more of these fascinating compounds.

## Experimental Section

**Computational details**: All calculations were undertaken by means of the program system TURBOMOLE,^30^ applying the TPSS functional^31^ and def2‐TZVP basis sets^32^ with the corresponding auxiliary bases^33^ and effective core potentials (ECPs) at In, Tl, Sn, Pb, Sb, and Bi.^34^ The electronic structures were investigated by Mulliken^10^ and natural population analyses (NPA),^11^ as well as by population analyses based on occupation numbers (Paboon)^12^ implemented in TURBOMOLE. COSMO, the conductor‐like screening model,^35^ was used to compensate the negative charges (standard values, *ϵ*=∞). Localized molecular orbitals were obtained via Boys’ method.^13^ The verification of the minima structures was done by analysis of the force constants.^36^ For more details, see the Supporting Information.

## Conflict of interest

The authors declare no conflict of interest.

## Supporting information

As a service to our authors and readers, this journal provides supporting information supplied by the authors. Such materials are peer reviewed and may be re‐organized for online delivery, but are not copy‐edited or typeset. Technical support issues arising from supporting information (other than missing files) should be addressed to the authors.

Supplementary
