# Supplementary material for: Systematic DFT Studies on Binary Pseudo‐tetrahedral Zintl Anions: Relative Stabilities and Reactivities towards Protons, Trimethylsilyl Groups, and Iron Complex Fragments
Source: Chemistry. 2020 Aug 17;26(51):11819–28. doi: 10.1002/chem.202001379 (PMC7540718; doi:10.1002/chem.202001379)
Supplement: Supplementary file 1 — Supplementary [file CHEM-26-11819-s001.pdf]

Chemistry–A European Journal

Supporting Information

Systematic DFT Studies on Binary Pseudo-tetrahedral Zintl Anions: Relative Stabilities and Reactivities towards Protons, Trimethylsilyl Groups, and Iron Complex Fragments

Lukas Guggolz and Stefanie Dehnen*[a]

1. Computational Details

All calculations were undertaken by employing the program system TURBOMOLE,[1] applying the TPSS functional[2] (grid size m3 or m4, where required) and the def2-TZVP basis set[3] with the corresponding auxiliary bases[4] and effective core potentials (ECPs) at In, Tl, Sn, Pb, Sb, and Bi.[5,6] The electronic structures were investigated by means of *Mulliken*[7] and natural population analyses (NPA),[8] as well as population analyses based on occupation numbers (PABOON)[9] as implemented in TURBOMOLE. COSMO, the conductor-like screening model,[10] was used to compensate the negative charges (standard values, $\varepsilon = \infty$). This way, all orbital energies of the occupied molecular orbitals remained negative during the optimizations. Localized molecular orbitals were obtained *via Boys*' method.[11]

All cluster geometries were first optimized with C_1 symmetry to find the global minimum structure. Where appropriate, they were afterwards again optimized with the respective higher symmetry. The verification of the minima structures was done by analysis of the force constants.[12–14]

The cluster structures were illustrated with the program DIAMOND 3.2k,[15] bond lengths and angles were analyzed with Molden.[16] The pictures of the canonical (MOs) and localized (LMOs) molecular orbitals were realized with gOpenMol.[17,18]

2. Calculated HOMO−LUMO Gaps

The following tables list the calculated orbital energies of the respective HOMOs and LUMOs and of the HOMO−LUMO gaps for all global minimum structures. Note, that all orbital energies of the occupied molecular orbitals remained negative during the optimizations.

Table S1. Calculated Orbital Energies of the HOMOs and LUMOs and the HOMO−LUMO Gaps for $(TrTt_3)^{5-}$.

	HOMO / eV	LUMO / eV	Gap / eV
$\mathbf{(AlSi_3)^{5-}}$	−2.27230	+0.32118	+2.59347
$\mathbf{(AlGe_3)^{5-}}$	−1.47899	+0.75832	+2.23731
$\mathbf{(AlSn_3)^{5-}}$	−2.30539	−0.24578	+2.05961
$\mathbf{(AlPn_3)^{5-}}$	−2.36093	−0.33422	+2.02671
$\mathbf{(GaSi_3)^{5-}}$	−2.18585	+0.49015	+2.67600
$\mathbf{(GaGe_3)^{5-}}$	−1.30589	+1.07910	+2.38499
$\mathbf{(GaSn_3)^{5-}}$	−2.25493	−0.21597	+2.03896
$\mathbf{(GaPb_3)^{5-}}$	−2.31467	−0.31936	+1.99531
$\mathbf{(InSi_3)^{5-}}$	−2.23504	+0.12017	+2.35520
$\mathbf{(InGe_3)^{5-}}$	−1.65883	+0.43277	+2.09159
$\mathbf{(InSn_3)^{5-}}$	−2.27922	−0.28039	+1.99883
$\mathbf{(InPb_3)^{5-}}$	−2.33248	−0.36451	+1.96797
$\mathbf{(TlSi_3)^{5-}}$	−2.17788	+0.19420	+2.37208
$\mathbf{(TlGe_3)^{5-}}$	−1.62894	+0.52732	+2.15626
$\mathbf{(TlSn_3)^{5-}}$	−2.23331	−0.25479	+1.97853
$\mathbf{(TlPb_3)^{5-}}$	−2.28719	−0.33299	+1.95420

Table S2. Calculated Orbital Energies of the HOMOs and LUMOs and the HOMO−LUMO Gaps for $(TrPn_3)^{2-}$.

	HOMO / eV	LUMO / eV	Gap / eV
$\mathbf{(AlP_3)^{2-}}$	−3.82419	−0.26676	+3.55743
$\mathbf{(AlAs_3)^{2-}}$	−3.65656	−0.49355	+3.16302
$\mathbf{(AlSb_3)^{2-}}$	−3.75331	−1.18360	+2.56971
$\mathbf{(AlBi_3)^{2-}}$	−3.61086	−1.16969	+2.44117
$\mathbf{(GaP_3)^{2-}}$	−3.67173	−0.19709	+3.47464
$\mathbf{(GaAs_3)^{2-}}$	−3.49869	−0.41246	+3.08624
$\mathbf{(GaSb_3)^{2-}}$	−3.64716	−1.14070	+2.50646
$\mathbf{(GaBi_3)^{2-}}$	−3.52134	−1.14354	+2.37780
$\mathbf{(InP_3)^{2-}}$	−3.51619	−0.48587	+3.03032
$\mathbf{(InAs_3)^{2-}}$	−3.38714	−0.53731	+2.84983
$\mathbf{(InSb_3)^{2-}}$	−3.56092	−1.16094	+2.39998
$\mathbf{(InBi_3)^{2-}}$	−3.45847	−1.15958	+2.29889
$\mathbf{(TlP_3)^{2-}}$	−3.33457	−0.43347	+2.90110
$\mathbf{(TlAs_3)^{2-}}$	−3.20834	−0.46428	+2.74406
$\mathbf{(TlSb_3)^{2-}}$	−3.43365	−1.12054	+2.31312
$\mathbf{(TlBi_3)^{2-}}$	−3.34391	−1.11821	+2.22570

Table S3. Calculated Orbital Energies of the HOMOs and LUMOs and the HOMO–LUMO Gaps for $(Tt_2Pn_2)^{2-}$.

	HOMO / eV	LUMO / eV	Gap / eV
**$(Si_2P_2)^{2-}$**	−3.65415	−0.21610	+3.43805
**$(Si_2As_2)^{2-}$**	−3.60593	−0.38489	+3.22104
**$(Si_2Sb_2)^{2-}$**	−3.79654	−1.06753	+2.72901
**$(Si_2Bi_2)^{2-}$**	−3.71993	−1.15575	+2.56419
**$(Ge_2P_2)^{2-}$**	−3.02591	+0.26185	+3.28776
**$(Ge_2As_2)^{2-}$**	−2.93897	+0.09791	+3.03688
**$(Ge_2Sb_2)^{2-}$**	−3.28110	−0.87805	+2.40305
**$(Ge_2Bi_2)^{2-}$**	−3.23697	−1.02859	+2.20838
**$(Sn_2P_2)^{2-}$**	−3.30761	−0.75527	+2.55234
**$(Sn_2As_2)^{2-}$**	−3.25292	−0.74312	+2.50980
**$(Sn_2Sb_2)^{2-}$**	−3.39144	−0.96924	+2.42220
**$(Sn_2Bi_2)^{2-}$**	−3.35413	−1.14762	+2.20651
**$(Pb_2P_2)^{2-}$**	−3.24466	−0.78223	+2.46244
**$(Pb_2As_2)^{2-}$**	−3.18442	−0.75200	+2.43242
**$(Pb_2Sb_2)^{2-}$**	−3.30282	−0.90762	+2.39520
**$(Pb_2Bi_2)^{2-}$**	−3.27073	−1.11419	+2.15654

Table S4. Calculated Orbital Energies of the HOMOs and LUMOs and the HOMO–LUMO Gaps for $(TrTt_3H_2)^{3-}$.

	HOMO / eV	LUMO / eV	Gap / eV
**$(AlSi_3H_2)^{3-}$***	−3.06803	−1.37795	+1.69008
**$(AlGe_3H_2)^{3-}$**	−2.50427	−0.66692	+1.83735
**$(AlSn_3H_2)^{3-}$**	−3.01645	−1.47673	+1.53972
**$(AlPb_3H_2)^{3-}$**	−3.01047	−1.43339	+1.57708
**$(GaSi_3H_2)^{3-}$**	−3.24599	−0.05742	+3.18857
**$(GaGe_3H_2)^{3-}$**	−2.48698	+0.35778	+2.84476
**$(GaSn_3H_2)^{3-}$**	−3.06643	−0.43284	+2.63359
**$(GaPb_3H_2)^{3-}$**	−3.07826	−0.53241	+2.54585
**$(InSi_3H_2)^{3-}$**	−3.15704	−0.50570	+2.65135
**$(InGe_3H_2)^{3-}$**	−2.59495	−0.24096	+2.35399
**$(InSn_3H_2)^{3-}$**	−3.03468	−0.50092	+2.53376
**$(InPb_3H_2)^{3-}$**	−3.03175	−0.58145	+2.45030
**$(TlSi_3H_2)^{3-}$**	−3.02747	−0.50913	+2.51834
**$(TlGe_3H_2)^{3-}$**	−2.54030	−0.22774	+2.31256
**$(TlSn_3H_2)^{3-}$**	−2.96051	−0.48680	+2.47371
**$(TlPb_3H_2)^{3-}$**	−2.96075	−0.51070	+2.45006

Note: All compounds marked with an asterisk (*) differ from the usual substitution pattern.

Table S5. Calculated Orbital Energies of the HOMOs and LUMOs and the HOMO−LUMO Gaps for ($TrPn_3H_2$).

	HOMO / eV	LUMO / eV	Gap / eV
**(AlP_3H_2)***	−5.76176	−1.95820	+3.80357
**($AlAs_3H_2$)**	−5.34281	−1.95288	+3.38992
**($AlSb_3H_2$)**	−5.01834	−2.29633	+2.72201
**($AlBi_3H_2$)**	−4.68723	−2.35186	+2.33538
**(GaP_3H_2)**	−5.75082	−1.91255	+3.83827
**($GaAs_3H_2$)**	−5.51450	−1.86244	+3.65206
**($GaSb_3H_2$)**	−5.21045	−2.10852	+3.10194
**($GaBi_3H_2$)**	−4.93862	−2.00533	+2.93329
**(InP_3H_2)**	−5.40764	−2.07040	+3.33724
**($InAs_3H_2$)**	−5.21788	−1.98058	+3.23729
**($InSb_3H_2$)**	−4.97363	−2.07522	+2.89840
**($InBi_3H_2$)**	−4.71891	−2.00521	+2.71370
**(TlP_3H_2)**	−5.19308	−2.07511	+3.11797
**($TlAs_3H_2$)**	−5.03393	−1.94192	+3.09201
**($TlSb_3H_2$)**	−4.83159	−2.01074	+2.82085
**($TlBi_3H_2$)**	−4.57617	−1.96672	+2.60945

Note: All compounds marked with an asterisk (*) differ from the usual substitution pattern.

Table S6. Calculated Orbital Energies of the HOMOs and LUMOs and the HOMO−LUMO Gaps for ($Tt_2Pn_2H_2$).

	HOMO / eV	LUMO / eV	Gap / eV
**($Si_2P_2H_2$)**	−6.02994	−3.68724	+2.34270
**($Si_2As_2H_2$)**	−5.82064	−3.62638	+2.19426
**($Si_2Sb_2H_2$)**	−5.43910	−2.78462	+2.65448
**($Si_2Bi_2H_2$)**	−5.19771	−2.80454	+2.39318
**($Ge_2P_2H_2$)**	−5.34665	−2.32793	+3.01871
**($Ge_2As_2H_2$)**	−5.90263	−2.70715	+3.19547
**($Ge_2Sb_2H_2$)**	−5.43535	−2.49229	+2.94306
**($Ge_2Bi_2H_2$)**	−5.19467	−2.49098	+2.70368
**($Sn_2P_2H_2$)**	−5.28660	−2.57906	+2.70754
**($Sn_2As_2H_2$)**	−5.25261	−2.50879	+2.74382
**($Sn_2Sb_2H_2$)**	−5.09904	−2.45681	+2.64224
**($Sn_2Bi_2H_2$)**	−4.99670	−2.48830	+2.50839
**($Pb_2P_2H_2$)**	−4.89847	−2.49287	+2.40560
**($Pb_2As_2H_2$)**	−4.88418	−2.42008	+2.46410
**($Pb_2Sb_2H_2$)**	−4.70282	−2.21196	+2.49086
**($Pb_2Bi_2H_2$)**	−4.63615	−2.25227	+2.38388

Table S7. Calculated Orbital Energies of the HOMOs and LUMOs and the HOMO–LUMO Gaps for $\{TrTt_3(SiMe_3)_2\}^{3-}$.

	HOMO / eV	LUMO / eV	Gap / eV
**$\{AlSi_3(SiMe_3)_2\}^{3-}$**	−2.83148	−1.03473	+1.79675
**$\{AlGe_3(SiMe_3)_2\}^{3-}$**	−2.31048	−0.02475	+2.28573
**$\{AlSn_3(SiMe_3)_2\}^{3-}$**	−2.79255	−1.27650	+1.51605
**$\{AlPb_3(SiMe_3)_2\}^{3-}$**	−2.78333	−1.26863	+1.51470
**$\{GaSi_3(SiMe_3)_2\}^{3-}$**	−2.82495	−0.07891	+2.74604
**$\{GaGe_3(SiMe_3)_2\}^{3-}$**	−2.46715	−0.11889	+2.34826
**$\{GaSn_3(SiMe_3)_2\}^{3-}$**	−2.79621	−1.05585	+1.74036
**$\{GaPb_3(SiMe_3)_2\}^{3-}$**	−2.80142	−1.08774	+1.71367
**$\{InSi_3(SiMe_3)_2\}^{3-}$**	−2.58503	−0.59230	+1.99272
**$\{InGe_3(SiMe_3)_2\}^{3-}$***	−2.34725	−0.40847	+1.93879
**$\{InSn_3(SiMe_3)_2\}^{3-}$**	−2.58463	−0.70304	+1.88159
**$\{InPb_3(SiMe_3)_2\}^{3-}$**	−2.86440	−1.12628	+1.73813
**$\{TlSi_3(SiMe_3)_2\}^{3-}$**	−2.50070	−0.59456	+1.90615
**$\{TlGe_3(SiMe_3)_2\}^{3-}$**	−2.19193	−0.36496	+1.82697
**$\{TlSn_3(SiMe_3)_2\}^{3-}$**	−2.52045	−0.65439	+1.86606
**$\{TlPb_3(SiMe_3)_2\}^{3-}$**	−2.70511	−0.47298	+2.23213

Note: All compounds marked with an asterisk (*) differ from the usual substitution pattern.

Table S8. Calculated Orbital Energies of the HOMOs and LUMOs and the HOMO–LUMO Gaps for $\{TrPn_3(SiMe_3)_2\}$.

	HOMO / eV	LUMO / eV	Gap / eV
**$\{AlP_3(SiMe_3)_2\}$**	−4.80119	−1.68410	+3.11709
**$\{AlAs_3(SiMe_3)_2\}$**	−4.79836	−1.81213	+2.98623
**$\{AlSb_3(SiMe_3)_2\}$**	−4.69632	−2.13314	+2.56318
**$\{AlBi_3(SiMe_3)_2\}$**	−4.53883	−2.09381	+2.44503
**$\{GaP_3(SiMe_3)_2\}$**	−4.77272	−1.84644	+2.92628
**$\{GaAs_3(SiMe_3)_2\}$**	−4.76026	−1.93413	+2.82613
**$\{GaSb_3(SiMe_3)_2\}$**	−4.66230	−2.14669	+2.51561
**$\{GaBi_3(SiMe_3)_2\}$**	−4.50023	−2.10577	+2.39445
**$\{InP_3(SiMe_3)_2\}$***	−4.39988	−1.95534	+2.44455
**$\{InAs_3(SiMe_3)_2\}$**	−4.50151	−2.06742	+2.43409
**$\{InSb_3(SiMe_3)_2\}$**	−4.46683	−2.18460	+2.28223
**$\{InBi_3(SiMe_3)_2\}$**	−4.33274	−2.17245	+2.16029
**$\{TlP_3(SiMe_3)_2\}$***	−4.16611	−2.03343	+2.13268
**$\{TlAs_3(SiMe_3)_2\}$***	−4.12238	−2.07477	+2.04761
**$\{TlSb_3(SiMe_3)_2\}$***	−4.20127	−1.97012	+2.23115
**$\{TlBi_3(SiMe_3)_2\}$**	−4.07421	−1.91609	+2.15812

Note: All compounds marked with an asterisk (*) differ from the usual substitution pattern.

Table S9. Calculated Orbital Energies of the HOMOs and LUMOs and the HOMO–LUMO Gaps for $\{Tt_2Pn_2(SiMe_3)_2\}$.

	HOMO / eV	LUMO / eV	Gap / eV
**$\{Si_2P_2(SiMe_3)_2\}$***	−4.94199	−1.54601	+3.39598
**$\{Si_2As_2(SiMe_3)_2\}$***	−4.83435	−1.38449	+3.44986
**$\{Si_2Sb_2(SiMe_3)_2\}$***	−4.64111	−1.65593	+2.98518
**$\{Si_2Bi_2(SiMe_3)_2\}$***	−4.41678	−1.76958	+2.64720
**$\{Ge_2P_2(SiMe_3)_2\}$**	−5.16762	−1.98768	+3.17994
**$\{Ge_2As_2(SiMe_3)_2\}$**	−5.19382	−2.75453	+2.43928
**$\{Ge_2Sb_2(SiMe_3)_2\}$**	−4.89306	−2.73064	+2.16242
**$\{Ge_2Bi_2(SiMe_3)_2\}$**	−4.65737	−2.61209	+2.04528
**$\{Sn_2P_2(SiMe_3)_2\}$**	−5.02088	−1.97192	+3.04895
**$\{Sn_2As_2(SiMe_3)_2\}$**	−5.05463	−2.08832	+2.96630
**$\{Sn_2Sb_2(SiMe_3)_2\}$**	−4.91727	−2.48753	+2.42974
**$\{Sn_2Bi_2(SiMe_3)_2\}$**	−4.61223	−2.76267	+1.84956
**$\{Pb_2P_2(SiMe_3)_2\}$**	−3.99472	−1.81205	+2.18267
**$\{Pb_2As_2(SiMe_3)_2\}$**	−4.73877	−2.03664	+2.70213
**$\{Pb_2Sb_2(SiMe_3)_2\}$***	−4.75224	−2.18607	+2.56617
**$\{Pb_2Bi_2(SiMe_3)_2\}$***	−4.59238	−2.12972	+2.46266

Note: All compounds marked with an asterisk (*) differ from the usual substitution pattern.

Table S10. Calculated Orbital Energies of the HOMOs and LUMOs and the HOMO–LUMO Gaps for $[\{CpFe(CO)_2\}_2(Tt_2Pn_2)]^{2-}$.

	HOMO / eV	LUMO / eV	Gap / eV
**$[\{CpFe(CO)_2\}_2(Si_2P_2)]^{2-}$**	−3.15759	−1.98365	+1.17394
**$[\{CpFe(CO)_2\}_2(Si_2As_2)]^{2-}$**	−3.25462	−2.01298	+1.24164
**$[\{CpFe(CO)_2\}_2(Ge_2P_2)]^{2-}$**	−2.72121	−1.78247	+0.93874
**$[\{CpFe(CO)_2\}_2(Ge_2As_2)]^{2-}$**	−2.81440	−1.83093	+0.98347
**$[\{CpFe(CO)_2\}_2(Sn_2P_2)]^{2-}$**	−2.90515	−1.87119	+1.03395
**$[\{CpFe(CO)_2\}_2(Sn_2As_2)]^{2-}$**	−2.98417	−1.93849	+1.04568
**$[\{CpFe(CO)_2\}_2(Pb_2P_2)]^{2-}$**	−2.87518	−1.83481	+1.04037
**$[\{CpFe(CO)_2\}_2(Pb_2As_2)]^{2-}$**	−2.93266	−1.90212	+1.03054

Table S11. Calculated Orbital Energies of the HOMOs and LUMOs and the HOMO–LUMO Gaps for $[\{CpFe(CO)_2\}_2(Tt_2Pn_2)]^{2-}$.

	HOMO / eV	LUMO / eV	Gap / eV
**$[Cu(NcMe)(Si_2P_2\{CpFe(CO_2\}_2]^{-}$**	−3.63139	−2.28957	+1.34182
**$[Cu(NcMe)(Si_2As_2\{CpFe(CO_2\}_2]^{-}$**	−3.80654	−2.34162	+1.46492
**$[Cu(NcMe)(Ge_2P_2\{CpFe(CO_2\}_2]^{-}$**	−3.24376	−2.02957	+1.21420
**$[Cu(NcMe)(Ge_2As_2\{CpFe(CO_2\}_2]^{-}$**	−3.48901	−2.19616	+1.29285
**$[Cu(NcMe)(Sn_2P_2\{CpFe(CO_2\}_2]^{-}$**	−3.32473	−2.21064	+1.11409
**$[Cu(NcMe)(Sn_2As_2\{CpFe(CO_2\}_2]^{-}$**	−3.46960	−2.29634	+1.17326
**$[Cu(NcMe)(Pb_2P_2\{CpFe(CO_2\}_2]^{-}$**	−3.21070	−2.13974	+1.07096
**$[Cu(NcMe)(Pb_2As_2\{CpFe(CO_2\}_2]^{-}$**	−3.34003	−2.25976	+1.08026

3. Comparison of Computationally Obtained and Experimental Data

Table S12. Comparison of Computationally Obtained and Experimental Data.

	Calculated / pm	Experimental / pm	Reference
**Sn1–Sn2**	294	292.30(30)	[19,20]
**Sn1–Sn3**	294	299.90(30)	
**Sn2–Sn3**	294	303.10(30)	
**Tl1–Sn1**	308	306.30(20)	
**Tl1–Sn2**	308	308.00(20)	
**Tl1–Sn3**	308	309.10(20)	
**Ga1–Bi1**	274	No experimental data provided by the authors.	[21]
**Ga1–Bi2**	274		
**Ga1–Bi3**	274		
**Bi1–Bi2**	295		
**Bi1–Bi3**	295		
**Bi2–Bi3**	295		
**Bi1–Bi2**	300	298.90(30) – 303.50(20)	[21]
**Bi1–Bi3**	300		
**Bi2–Bi3**	300		
**In1–Bi1**	308		
**In1–Bi2**	308		
**In1–Bi3**	308		
**Bi1–Bi2**	300	304.59(8) – 307.72(6)	[22]
**Bi1–Bi3**	300		
**Bi2–Bi3**	300		
**Tl1–Bi1**	315		
**Tl1–Bi2**	315		
**Tl1–Bi3**	315		
**P1–P2**	223	238.70(20) – 263.50(20)	[23]
**Ge1–P1**	241		
**Ge1–P2**	241		
**Ge1–P1**	241		
**Ge2–P2**	241		
**Ge1–Ge2**	255		
**As1–As2**	245	245.90(10) – 257.20(10)	[24]
**Ge1–As1**	252		
**Ge1–As2**	252		
**Ge2–As1**	252		
**Ge2–As2**	252		
**Ge1–Ge2**	254		
**Sb1–Sb2**	285	285.18(9) – 288.39(6)	[25]
**Sn1–Sb1**	291		
**Sn1–Sb2**	291		
**Sn2–Sb1**	291		
**Sn2–Sb2**	291		
**Sn1–Sn2**	293		
**Sn1–Sn2**	293	287.00(8)	[26]
**Sn1–Bi1**	299	299.23(8)	(see also [27,28])
**Sn1–Bi2**	299	298.80(8)	
**Sn2–Bi1**	299	298.15(10)	
**Sn2–Bi2**	299	298.57(9)	
**Bi1–Bi2**	301	303.16(6)	
**Sb1–Sb1**	285	291.80(30)	[29]
**Sb1–Pb1**	299	293.10(30)	
**Sb1–Pb2**	299	292.40(20)	
**Sb2–Pb1**	299	297.40(50)	
**Sb2–Pb2**	299	296.30(50)	
**Pb1-Pb2**	308	300.60(40)	
**Bi1–Bi2**	301	301.30(20) – 304.80(20)	[30]
**Bi1–Pb1**	306		
**Bi1–Pb2**	306		
**Bi2–Pb1**	306		
**Bi2–Pb2**	306		
**Pb1–Pb2**	308		

Based on these studies, we are able to better understand the structures, which are afflicted by complete or partial disorder in the X-ray structures of their respective salts. For example, *Xu* and *Sevov* reported bond lengths in the range of 298.90(30)–303.50(20) pm within the anion $(InBi_3)^{2-}$.[21] Our calculations showed that the Bi–Bi bond lengths are the smaller ones with 300 pm, while the In–Bi bonds are slightly longer and yield 308 pm. Similarly, our studies confirmed the assumption made by *Critchlow* and *Corbett*, that the shortest bond in the $(Pb_2Sb_2)^{2-}$ anion is the Sb–Sb bond, while the Pb–Pb bond is the longest within this cluster. As expected, the Pb–Sb bonds are somewhere in between.[29]

4. Canonical Molecular Orbitals of $(InGe_3)^{5-}$

Figure S1 shows the interchanged sequence of the canonical frontier orbitals in some of the group 13/14 *pseudo*-tetrahedral anions compared to the regular order shown in Figure 4 of the manuscript. $(InGe_3)^{5-}$ is given here as an example.

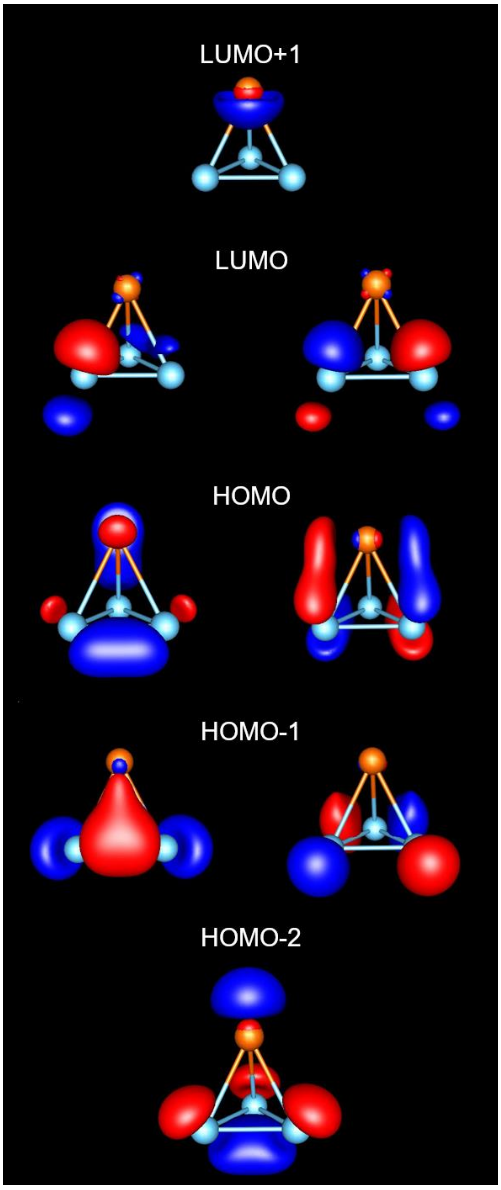

Figure S1. Canonical MOs of $(InGe_3)^{5-}$ (In: orange, Ge: sky blue; contour values: ±0.05 a. u.).

5. Silylation Pattern of {$TrSi_3(SiMe_3)_2$} (with Tr: Al, Ga, In, Tl)

{$GaSi_3(SiMe_3)_2$} is given as an example for the alternative silylation pattern (Figure S2):

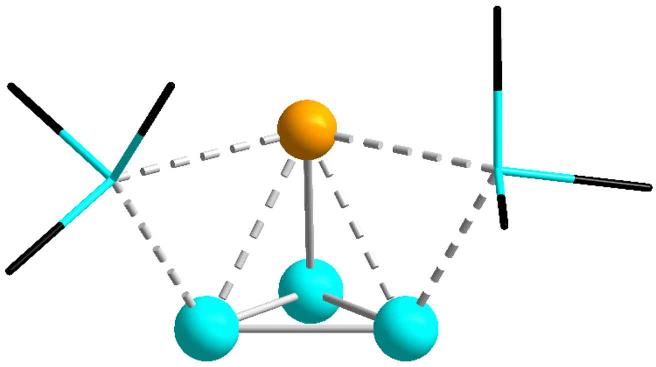

Figure S2. Calculated minimum structure of {$GaSi_3(SiMe_3)_2$} (Ga: orange, Si: turquoise, C: black, H atoms are omitted for clarity). Selected bond lengths (pm): Ga–Si_{core} 247 and 269, Si_{core}–Si_{core} 237 and 242, Ga–Si_{TMS} 286 and 292, Si_{core}–Si_{TMS} 242 and 247.

{$GaSi_3(SiMe_3)_2$} and its heavier homologues show a slightly more asymmetric silylation pattern due to the steric repulsion between the two TMS groups.

6. Silylation Pattern of {$TlPn_3(SiMe_3)_2$} (with Pn: P, As, Sb)

{$TlP_3(SiMe_3)_2$} is given as an example for the alternative silylation pattern (Figure S3):

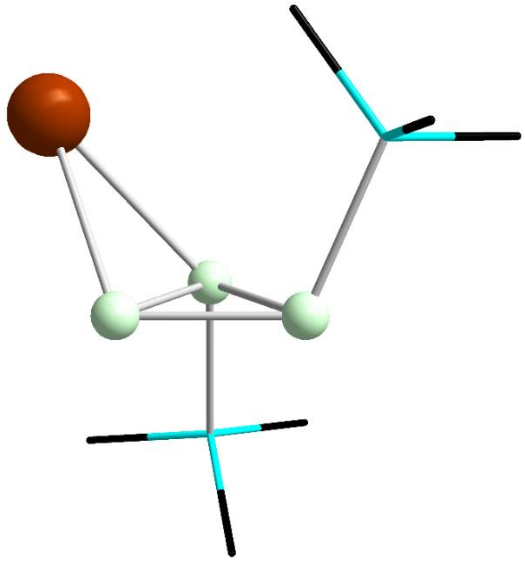

Figure S3. Calculated minimum structure of {$TlP_3(SiMe_3)_2$} (Tl: brown, P: light green, C: black, H atoms are omitted for clarity). Selected bond lengths (pm): Tl–P 281 and 296; P–P 220, 221, and 224; P–Si_{TMS} 229.

One of the Tl–P bonds of the former *pseudo*-tetrahedron is broken, which yields in the formation of butterfly-like anionic core.

7. Silylation Pattern of {$Pb_2Pn_2(SiMe_3)_2$} (with Pn: Sb, Bi)

{$Pb_2Bi_2(SiMe_3)_2$} is given as an example for the alternative silylation pattern (Figure S4):

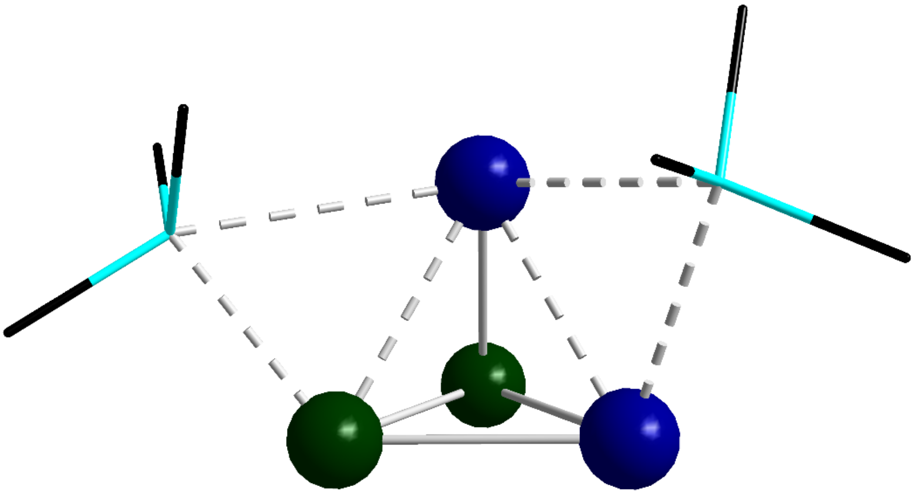

Figure S4. Calculated minimum structure of {$Pb_2Bi_2(SiMe_3)_2$} (Pb: dark blue, Bi: dark green, Si: turquoise, C: black, H atoms are omitted for clarity). Selected bond lengths (pm): Pb–Pb 320; Pb–Bi 297, 306, 307, and 321; As–As 301; Pb–Si_{TMS} 297, 303, and 345; As–Si_{TMS} 276.

8. Silylation Pattern of {$Si_2Pn_2(SiMe_3)_2$} (with Pn: P, As, Sb, Bi)

{$Si_2As_2(SiMe_3)_2$} is given as an example for the alternative silylation pattern (Figure S5):

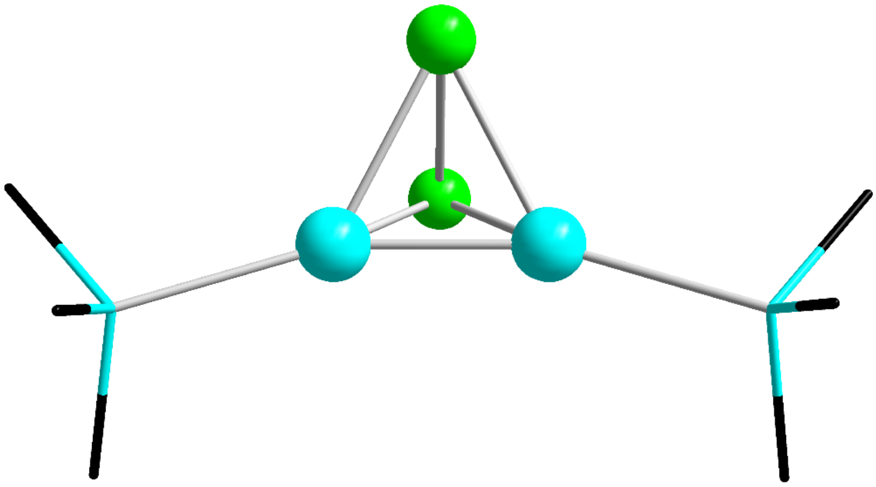

Figure S5. Calculated minimum structure of {$Si_2As_2(SiMe_3)_2$} (Si: turquoise, As: bright green, C: black, H atoms are omitted for clarity). Selected bond lengths (pm): As–Si 239; Si_{core}–Si_{core} 222; As–As 251; Si_{core}–Si_{TMS} 236.

9. Canonical Molecular Orbitals of $[\{CpFe(CO)_2\}_2(Ge_2P_2)]^{2-}$

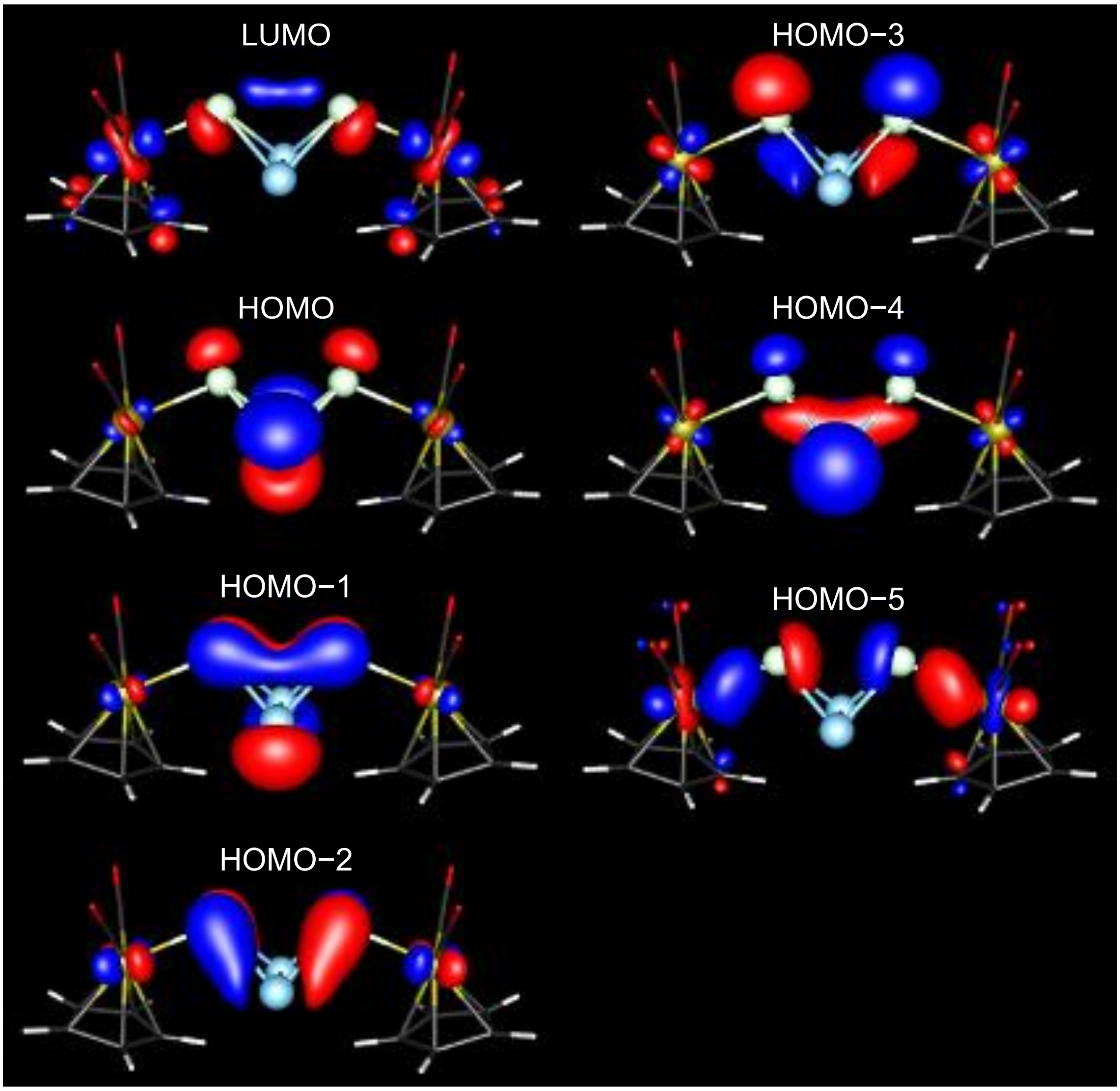

Figure S6. Canonical MOs of $[\{CpFe(CO)_2\}_2(Ge_2P_2)]^{2-}$ (Ge: sky blue, P: light green, Fe: dark yellow, O: red, C: black, H: grey; contour values: ±0.05 a. u.).

10. Calculated Reaction Energies

Table S13. Calculated Reaction Energies for the Hypothetical Reaction of the Butterfly-like Anions With a $[Cu(NCMe)]^+$ Fragment

Complex	Relative Energy / kJ·mol^{-1}
$[Cu(NCMe)(Si_2P_2\{CpFe(CO)_2\}_2)]^-$	−342
$[Cu(NCMe)(Si_2As_2\{CpFe(CO)_2\}_2)]^-$	−337
$[Cu(NCMe)(Ge_2P_2\{CpFe(CO)_2\}_2)]^-$	−358
$[Cu(NCMe)(Ge_2As_2\{CpFe(CO)_2\}_2)]^-$	−350
$[Cu(NCMe)(Sn_2P_2\{CpFe(CO)_2\}_2)]^-$	−349
$[Cu(NCMe)(Sn_2As_2\{CpFe(CO)_2\}_2)]^-$	−334
$[Cu(NCMe)(Pb_2P_2\{CpFe(CO)_2\}_2)]^-$	−351
$[Cu(NCMe)(Pb_2As_2\{CpFe(CO)_2\}_2)]^-$	−338

11. References for the Supporting Information

[1] TURBOMOLE V6.6/7.0.1/7.1.1 2014/2015/2016, a development of University of Karlsruhe and Forschungszentrum Karlsruhe GmbH, 1989–2007, TURBOMOLE GmbH, since 2007; available from http://www.turbomole.com.

[2] J. Tao, J. P. Perdew, V. N. Staroverov, G. E. Scuseria, *Phys. Rev. Lett.* **2003**, *91*, 3–6.

[3] F. Weigend, R. Ahlrichs, *Phys. Chem. Chem. Phys.* **2005**, *7*, 3297–3305.

[4] F. Weigend, *Phys. Chem. Chem. Phys.* **2006**, *8*, 1057–1065.

[5] B. Metz, H. Stoll, M. Dolg, *J. Chem. Phys.* **2000**, *113*, 2563–2569.

[6] B. Metz, M. Schweizer, H. Stoll, M. Dolg, W. Liu, *Theor. Chem. Acc.* **2000**, *104*, 22–28.

[7] R. S. Mulliken, *J. Chem. Phys.* **1955**, *23*, 2338–2342.

[8] A. E. Reed, R. B. Weinstock, F. Weinhold, *J. Chem. Phys.* **1985**, *83*, 735–746.

[9] C. Ehrhardt, R. Ahlrichs, *Theor. Chim. Acta* **1985**, *68*, 231–245.

[10] A. Klamt, G. Schüürmann, *J. Chem. Soc., Perkin Trans. 2* **1993**, *2*, 799–805.

[11] S. F. Boys, in *Quantum Theory of Atoms, Molecules and the Solid State* (Ed.: P.-O. Löwdin), New York, **1966**, pp. 253–262.

[12] P. Deglmann, F. Furche, R. Ahlrichs, *Chem. Phys. Lett.* **2002**, *362*, 511–518.

[13] P. Deglmann, F. Furche, *J. Chem. Phys.* **2002**, *117*, 9535–9538.

[14] P. Deglmann, K. May, F. Furche, R. Ahlrichs, *Chem. Phys. Lett.* **2004**, *384*, 103–107.

[15] Diamond – Crystal and Molecular Structure Visualization, Crystal Impact – Dr. H. Putz & Dr. K. Brandenburg GbR, Kreuzherrenstr. 102, 53227 Bonn, Germany, http://www.crystalimpact.com/diamond.

[16] G. Schaftenaar, J. H. Noordik, *J. Comput. Aided. Mol. Des.* **2000**, *14*, 123–134.

[17] L. Laaksonen, *J. Mol. Graph.* **1992**, *10*, 33–34.

[18] D. L. Bergman, L. Laaksonen, A. Laaksonen, *J. Mol. Graph. Model.* **1997**, *15*, 301–306.

[19] W. Blase, G. Cordier, *Z. Krist.* **1990**, *193*, 207–211.

[20] W. Blase, G. Cordier, *Z. Krist.* **1991**, *196*, 319–320.

[21] L. Xu, S. C. Sevov, *Inorg. Chem.* **2000**, *39*, 5383–5389.

[22] N. Lichtenberger, N. Spang, A. Eichhöfer, S. Dehnen, *Angew. Chem. Int. Ed.* **2017**, *56*, 13253–13258.

[23] S. Mitzinger, J. Bandemehr, K. Reiter, J. Scott McIndoe, X. Xie, F. Weigend, J. F. Corrigan, S. Dehnen, *Chem. Commun.* **2018**, *54*, 1421–1424.

[24] S. Mitzinger, L. Broeckaert, W. Massa, F. Weigend, S. Dehnen, *Nat. Commun.* **2016**, *7*, 10480.

[25] F. Lips, I. Schellenberg, R. Pöttgen, S. Dehnen, *Chem. Eur. J.* **2009**, *15*, 12968–12973.

[26] U. Friedrich, M. Neumeier, C. Koch, N. Korber, *Chem. Commun.* **2012**, *48*, 10544–10546.

[27] S. C. Critchlow, J. D. Corbett, *Inorg. Chem.* **1982**, *21*, 3286–3290.

[28] F. Lips, M. Raupach, W. Massa, S. Dehnen, *Z. Anorg. Allg. Chem.* **2011**, *637*, 859–863.

[29] S. C. Critchlow, J. D. Corbett, *Inorg. Chem.* **1985**, *24*, 979–981.

[30] R. Ababei, J. Heine, M. Hołyńska, G. Thiele, B. Weinert, X. Xie, F. Weigend, S. Dehnen, *Chem. Commun.* **2012**, *48*, 11295–11297.
